# *Hyssopus cuspidatus* volatile oil: a potential treatment for steroid-resistant asthma via inhibition of neutrophil extracellular traps

**DOI:** 10.1186/s13020-025-01069-2

**Published:** 2025-02-03

**Authors:** Xu Wang, Hui-Ming Peng, Meng-Ru Zhang, Jing-Jing Li, Chuan-Peng Zhao, Ya-Li Zhang, Si-Yu Wang, Si-Ying Zhu, Jian-Kang Lu, Hai-Long Yin, Qiang Yin, Jin-Bo Fang

**Affiliations:** 1 School of Pharmacy, Hubei Key Laboratory of Natural Medicinal Chemistry and Resource Evaluation, Tongji Medical College, Huazhong University of Science and Technology, Wuhan, 430030 China; 2 Department of Anatomy, Tongji Medical College, Huazhong University of Science and Technology, Wuhan, 430030 China; 3 Hubei Shizhen Laboratory, School of Basic Medicine, Hubei University of Chinese Medicine, Wuhan, 430065 China; 4 Xinjiang Uygur Pharmaceutical Co., Ltd, No. 2, Shenyang Street, Urumqi Economic and Technological Development Zone (Toutunhe District), Xinjiang Uygur Autonomous Region, Urumqi, 830026 Xinjiang China

**Keywords:** *Hyssopus cuspidatus* Boriss., Volatile oil, Steroid-resistant asthma, Neutrophil extracellular traps, Neutrophil elastase

## Abstract

**Background:**

Steroid-resistant asthma (SRA) is a form of asthma resistant to corticosteroid therapy, which is characterized by the presence of neutrophil-predominant inflammatory response and neutrophil extracellular traps (NETs) formation. *Hyssopus cuspidatus* Boriss., a traditional Uyghur medicine, is known for its efficacy in treating inflammatory lung conditions such as asthma. However, the therapeutic impact and underlying mechanisms of *Hyssopus cuspidatus* Boriss.’s volatile oil (HVO) in SRA have not been fully elucidated.

**Methods:**

This study established an ovalbumin/lipopolysaccharide (OVA/LPS)-induced SRA mice model to evaluate the therapeutic effect of HVO on SRA. UPLC-QE-Orbitrap-MS was applied to analyze the serum compositions of HVO. Network pharmacology and molecular docking were employed to uncover the complex mechanisms of HVO in treating SRA and predict potential effective compounds in HVO. Furthermore, in vivo studies in SRA mice and in vitro studies using HL-60 cells and bone marrow neutrophils were conducted to validate the mechanism.

**Results:**

HVO could significantly ameliorate OVA/LPS-induced SRA symptoms, including airway hyperresponsiveness, airway inflammation, mucus overproduction and airway remodeling. 41 prototype compounds, 65 Phase I metabolites and 50 Phase II metabolites were identified in serum-containing HVO. The integration of network pharmacology with experimental validation revealed that HVO can inhibit the formation of NETs by targeting neutrophil elastase, thereby exerting a therapeutic influence on SRA. Meanwhile, molecular docking results showed that 3-methoxy-4-hydroxy mandelonitrile, 1,2,3,4-tetrahydro-1,5,7-trimethyl-naphthalene, cis-calamenene and aristol-1(10)-en-9-yl isovalerate may be the therapeutic compounds of HVO in treating SRA.

**Conclusion:**

These findings suggest that HVO is a promising therapeutic candidate for neutrophil-dominant SRA by targeting NETs formation.

**Supplementary Information:**

The online version contains supplementary material available at 10.1186/s13020-025-01069-2.

## Introduction

Asthma is a chronic respiratory disease characterized by airway hyper-responsiveness (AHR), airway inflammation and remodeling, and mucus overproduction, resulting in symptoms such as recurrent wheezing, shortness of breath, chest tightness, cough, and expiratory airflow limitation [1]. The prevalent phenotype of asthma is allergic asthma, which is associated with type 2 eosinophilic responses, and anti-inflammatory inhaled corticosteroids are the mainstay of treatment [2]. However, some patients do not respond well to high-dose inhaled corticosteroids and they are considered to have steroid-resistant asthma (SRA), which is associated with frequent exacerbations and airflow limitations [3]. Clinical trials have revealed that patients with SRA often show neutrophil-predominant inflammatory response, essentially the formation of neutrophil extracellular traps (NETs), with the presence of T helper cell 17 and cytokines including IL-17, IL-1β, IL-8, and IFN-γ [4, 5]. NETs are weblike DNA structures coated with histones and antimicrobial proteins such as neutrophil elastase (NE), citrullinated histone3 (citH3) and myeloperoxidase (MPO), and cause damage to host tissue [6]. Upon exposure to various triggers such as allergens, viral and bacterial components, smoking and inflammatory signals, the epithelial cells and macrophages respond by generating chemokines CXCL1/2, IL-1β, IL-6, and IL-8. These chemokines induced neutrophils chemotaxis and infiltration into the airways and the release of NETs [7]. Moreover, IL-17 produced from Th17 cells recruits neutrophils to the airway and promotes neutrophilic inflammation and steroid resistance [8]. During the processes of NETs formation, mediators released from neutrophils, such as reactive oxygen species (ROS) and NE, help in host defense and pathogen elimination, but they are known to promote bronchoconstriction, contribute to increased mucus production, cause collateral damage to the lungs and exacerbate airway diseases [4].

The prevalence of SRA has been reported to be 10% of the overall population with asthma and represents more than 50% of the asthma-associated healthcare burden [9]. According to the 2024 Global Strategy for Asthma Management and Prevention by the Global Initiative for Asthma, combination therapy by long-acting beta_2_-agonist and high-dose inhaled corticosteroids together with long-acting muscarinic antagonists is recommended to improve lung function and reduce exacerbations of SRA at present (https://ginasthma.org/). Unfortunately, the combined use of long-acting beta_2_-agonist and inhaled corticosteroids has been reported to have significant side effects, such as enhancing the sensitivity of cardiac beta_2_-adrenoceptors, thereby inducing arrhythmias [10]. Current researches on SRA treatment focus on chemical drugs, hence it is urgent to develop a new therapy for SRA on traditional Chinese medicine to optimize a balance between safety, efficacy, and cost. Particularly, the development of single medicine that can address the challenges in the management of SRA by simultaneously improving airway hyper-responsiveness, airway inflammation and remodeling is promising.

Uyghur medicine, has attracted attentions from the medical and pharmaceutical communities in recent years [11]. One of the representative formulas of Uyghur medicine, Hanchuan Zupa granule, has been approved by the National Medical Products Administration of China for clinical treatment of asthma (National Drug Approval Letter Z20063931). *Hyssopus cuspidatus* Boriss. is the main component of the Hanchuan Zupa granules and it is also used to treat damp-cold respiratory diseases [12]. *Hyssopus cuspidatus* volatile oil (HVO) contain active constituents such as β-pinene, pinocamphone, and isopinocamphone. The herbal drug is generally regarded as safe and possesses anti-inflammatory, antitussive, expectorant, and other biological activities [13]. Our previous study have shown that HVO was effective in treating allergic asthma without observable toxic effects on the major organs [14]. However, its effects on neutrophil-dominant SRA and the underlying mechanisms remain unclear.

In the present study, we evaluated the therapeutic potential of HVO in OVA/LPSinduced SRA mice model. To throw light on its underlying mechanisms, UPLC-QE-Orbitrap-MS analysis of the serum of HVO-treated rats was performed to detect the HVO metabolites, and molecular docking was applied to predict the therapeutic compounds. The pharmacological mechanism of HVO in treating SRA was predicted using network pharmacology and experimentally validated via in vivo and in vitro study. These findings provide a research foundation for the development of HVO as a therapeutic agent for SRA.

## Materials and methods

### Extraction of Hyssopus cuspidatus volatile oil

*H. cuspidatus* was collected from Tacheng City, Xinjiang Uygur Autonomous Region of China and identified by Prof. Jin-Bo Fang at Huazhong University of Science and Technology, Wuhan, China. A voucher herbarium specimen has been deposited at the affiliated Pharmacognosy Laboratory (No. 20,191,001). The air-dried *H. cuspidatus* was crushed and soaked in distilled water overnight, followed by continuous extraction for 9 h in a volatile oil extractor. HVO was obtained with anhydrous sodium sulfate, and stored at 4 °C protected from light.

### Animals

Female BALB/c mice (6 weeks, 20 ± 2 g body weight) were purchased from Beijing SiPeiFu Biotechnology Co., Beijing, China [SYXK (Jing) 2019-0010] and housed under Individual Ventilated Cages conditions. Pathogen-free male Sprague–Dawley rats (6 weeks, 200 ± 20 g body weight) and male C57BL/6 mice (10 weeks, 20 ± 2 g body weight) were purchased from Hubei Biont Biological Technology Co., Wuhan, China [SYXK (E) 2021-0027]. All animals were raised at the Experimental Animal Center of the Huazhong University of Science and Technology (HUST) and provided with standard rodent chow and water ad libitum under controlled environment (25 ± 2 °C; 60 ± 5%; 12 h light dark cycle). The experimental protocol was approved by the Animal Ethics Committee at Tongji Medical College of HUST (IACUC No. 3674).

### Preparation of HVO-containing serum

After 1 week of adaptive feeding, the Sprague–Dawley rats were randomly divided into two groups (n = 4). The control group received 0.5% sodium carboxymethyl cellulose and the HVO-group received 2.4 mg/kg of HVO suspended in 0.5% sodium carboxymethyl cellulose solution (fivefold the clinically equivalent dose of HVO used in Uygur medicine). All drugs were administered via gavage for three consecutive days, twice a day. Consequently, rats were administered a daily dosage of 4.8 mg/kg of HVO (tenfold the clinical equivalent dose). One-hour after the last administration, rats were anesthetized by intraperitoneal injection of 1% pentobarbital sodium, and blood samples were collected from the abdominal aortas and centrifuged at 3000 rpm for 15 min. The supernatant was inactivated at 56 °C for 30 min filtered using a 0.22 μm membrane filter and then stored at − 80 °C until use [15].

### Ingredients identification of the serum of HVO-treated rats by UPLC-QE-Orbitrap-MS

The serum (200 μL) was treated with 1200 μL acetonitrile (Sinopharm Chemical Reagent Co. China) and vortexed for 30 s. After centrifuging at 13,000 rpm (4 °C) for 15 min, 1300 μL of supernatant was dried under nitrogen. The residue was re-dissolved in 200 μL methanol (Sinopharm Chemical Reagent Co. China), vortexed for 30 s and centrifuged at 13,000 rpm (4 °C) for 15 min. The supernatant was analyzed by UPLC-QE-Orbitrap-MS using an Ultimate 3000 UPLC coupled with a high-resolution Q Exactive Orbitrap tandem mass spectrometer (Thermo Fisher Scientific Inc., Germany). Chromatographic separation was performed on a Hypersil GOLD column (100 × 2.1 mm, 3 μm) at 25 °C. The mobile phase consisted of acetonitrile (A) and 0.1% (v/v) formic acid (Sigma Aldrich) aqueous solution (B), and the gradient program was set as follows: 0–2 min, 5% A; 2–14 min, 5–95% A; 14–19 min, 95% A; 19–19.1 min, 95–5% A; and 19.1–25 min, 5% A. The flow rate was 0.25 mL/min, and the injection volume was 5 μL. The mass spectrometer was operated in both positive and negative ion modes, and the mass range was set to 100–1500 Da. The MS parameters were set as follows: ion source, electrospray ionization; spray voltage, 3200 V; capillary temperature, 300 °C; sheath gas, 40.00 Arb; aux gas, 8.00 Arb; max spray current, 100 µA; probe heater temperature, 275 °C. Data analysis was performed by the Xcalibur v4.1.50 software (Thermo Fisher Scientific Inc.), and compound identity was established by comparing the retention time, mass and fragment ions with literature values.

### Pathways prediction based on network pharmacology

Targets of the 41 prototype components in HVO-containing serum were predicted using the SwisstargetPrediction database (http://swisstargetprediction.ch/) and normalized by UniProt (https://www.uniprot.org/). Targets associated with SRA were searched from Genecard (https://www.genecards.org/) and OMIM (https://omim.org/) databases, using the keyword “steroid resistant asthma”. The protein-protein interaction network was created by STRING database (https://cn.string-db.org/) and visualized using Cytoscape 3.9.1 software. The topological parameters in the network were calculated using Network Analyzer software plug-in for Cytoscape. Gene Ontology and Kyoto Encyclopedia of Genes and Genomes enrichment analyses were used for functional annotations and performed in the DAVID database (https://david.ncifcrf.gov/).

### Molecular docking

The PDB database (https://www1.rcsb.org/) was searched to obtain molecular structure files of the protein targets MPO (PDB ID: 5MFA), NE (PDB ID: 5ABW), IL-17 (PDB ID: 8USS) and IL-8 (PDB ID: 4XDX). 2D-structures of the 41 prototype compounds identified in HVO-containing serum as ligands were obtained from the PubChem database (https://pubchem.ncbi.nlm.nih.gov/), and Chem3D software was applied to optimize molecular mechanics for the optimal conformations and conversion into 3D-structures. Subsequently, the Auto Dock Vina v.1.2.0 was used to calculate the affinity between protein targets and the ligands. Finally, the PyMOL software and Discovery Studio 2016 Client were used to visualize the molecular docking results.

### SRA mouse model and drug treatment

BALB/c mice were randomly divided into the following groups (n = 5): Control, OVA/LPS, OVA/LPS + Dexamethasone (Dexa), OVA/LPS + Sivelestat (SIV), OVA/LPS + low dose of HVO (HVO-Low), OVA/LPS + middle dose of HVO (HVO-Mid), OVA/LPS + high dose of HVO group (HVO-High), OVA/LPS + SIV + middle dose of HVO group (HVO + SIV). Mice were sensitized by intraperitoneal injection of a mixture of OVA (Aladdin; 1.5 mg/kg) and Al(OH)_3_ adjuvant (Sigma Aldrich; 50 mg/kg) on days 0, 7, and 14, and stimulated intranasally with LPS (LPS from *Escherichia coli* 0111: B4, Sigma Aldrich; 0.5 mg/kg) on day 21. On days 22–26, the mice were challenged by inhalation of aerosolized 1% OVA for 30 min once a day. For drug treatment, dexamethasone (Sigma Aldrich; 5 mg/kg, i.p.), SIV (Yuanye, Shanghai, China; 100 mg/kg, i.p.) and HVO-Low (0.85 mg/kg, i.g.), HVO-Mid (1.71 mg/kg, i.g.), or HVO-High (3.42 mg/kg, i.g.) were administered 30 min before each OVA challenge, respectively. The HVO doses were determined based on the conversion formula between experimental animals and humans, 1.71 mg/kg of HVO being equivalent to the clinical dose in humans.

### Measurement of airway hyper-responsiveness

To evaluate lung function, AHR was measured 24 h after the last challenge. The mice were anesthetized intraperitoneally with 1% pentobarbital sodium, followed by tracheotomy and intubation. Subsequently, the inserted tracheal tube was connected to the ventilator of a FlexiVent system (SCIREQ, Montreal, QC, Canada). Increasing doses of methacholine (0, 3.125, 6.25, 12.5, 25, and 50 mg/mL; Sigma Aldrich) were nebulized using an ultrasonic nebulizer connected to the FlexiVent system to induce bronchoconstriction. Individual peak response values of resistive resistance were recorded after each nebulization for lung function measurement.

### Inflammatory cell count

Twenty-four hours after the last challenge, the mice were anesthetized intraperitoneally with 1% pentobarbital sodium. Peripheral blood samples were collected and treated with anticoagulants. Bronchoalveolar lavage fluid (BALF) was also collected and divided into two parts. The first was used for cytokine analysis, and the second was applied in cell counting using a hemocytometer under light microscope. Differential cell counts in the peripheral blood and BALF were determined using Wright–Giemsa staining. Relative percentages of eosinophils and neutrophils were recorded.

### Enzyme-linked immunosorbent assay

Peripheral blood samples and the first BALF were centrifuged at 3500 rpm for 15 min and 2000 rpm for 10 min at 4 °C, respectively. The supernatant was stored at − 80 °C for cytokine measurement. Lungs were isolated and homogenized in phosphate-buffered saline, and the homogenate was centrifuged at 6000 × *g* for 10 min at 4 °C. The IL-17 (Mlbio), IL-8 (Mlbio), CXCL1 (Elabscience), and CXCL2 (Elabscience) levels in the BALF, serum, and lung tissues were determined using commercial ELISA kits according to the manufacturer’s protocol.

### Lung histology

The left lung lobes of the mice were fixed in 4% paraformaldehyde, paraffin-embedded and 7-µm sections were cut. The extent of cell infiltration and mucus production in the lungs was evaluated using hematoxylin and eosin and periodic acid-Schiff staining, respectively.

### Western blot

The lung tissues were lysed in ice-cold RIPA lysis buffer containing protease (Sigma Aldrich) and phosphatase inhibitors (Sigma Aldrich) at 4 °C for 30 min, followed by centrifugation at 14,000 g for 30 min to collect the protein supernatant. Protein content was determined by bicinchoninic acid assay (Biosharp). The samples were then separated on SDS-PAGE and transferred to PVDF membranes (Sigma Aldrich). After blocked with 5% skim milk for 2 h, the membranes were incubated with rabbit primary antibodies at 4 °C overnight: p-JNK (Beyotime), JNK (Beyotime), p-P38 (Absin), P38 (Beyotime), p-ERK (Cell Signaling Technology) and β-actin (Abbkine), ERK (Beyotime). The membranes were washed in TBST and incubated with anti-rabbit secondary antibodies (DaiAn) for 1 h at room temperature, and then washed and photographed using an imaging analysis system (GE Healthcare, Little Chalfont, UK). The mean grey values were analyzed and normalized by ImageJ software.

### Immunohistochemistry staining

After de-paraffin, antigen retrieval, being blocked endogenous peroxidase nonspecific binding, lung tissue slides were incubated with anti-MPO (1:100; Beyotime) antibody, anti-citH3 antibody (1:200; Abcam), and anti-NE antibody (1:200; Abcam) at 4 °C overnight. After wash, the slides were incubated with horseradish peroxidase-conjugated goat anti-rabbit IgG (Bosterbio) for 1 h at room temperature and stained with diaminobenzidine (ZSGB-BIO). Cell nuclei were counterstained with hematoxylin. Images were captured under light microscope, followed by analysis using the ImageJ software.

### Isolation of mice neutrophils and differentiated HL-60 cell culture

Bone marrow cells from the femurs and tibias of the male C57BL/6 mice were flushed in sterile phosphate-buffered saline, and filtered through a 70 µm cell strainer. Neutrophils were then separated from bone marrow cells by plating 1 mL of the cell suspension onto a Percoll (Biosharp) gradient consisting of 3 mL of 78% Percoll, 3 mL of 62.5% Percoll, 2 mL of 55% Percoll to 2 mL of 55% Percoll, followed by centrifugation at 900 × g for 30 min at 10 °C. The neutrophil layer between 78% and 62.5% Percoll was collected, washed twice with phosphate-buffered saline, and resuspended in serum-free RPMI 1640 medium.

The HL-60 cell line was obtained from the Cell Bank of Wuhan University, China Center for Type Culture Collection (CCTCC; Wuhan, China). HL-60 cells were cultured in an RPMI-1640 medium containing 10% FBS, streptomycin (100 U/mL), and penicillin (100 U/mL), and maintained in an incubator in a humidified atmosphere containing 5% CO_2_ at 37 °C. Neutrophil-like differentiated HL-60 were induced by adding all-trans-retinoic acid (Sigma Aldrich; 0.1, 1, or 10 µM) to the culture media. Giemsa staining and trypan blue (0.08%) staining were used to detect the differentiation effects under different concentration of all-trans-retinoic acid and induction time.

### Flowcytometry analyses of differentiated HL-60 cells

Single-cell suspension of differentiated HL-60 cells as described above was obtained. Cells were dyed with flow cytometric antibodies for neutrophils (human: CD11b + CD15 +). Samples were analyzed on a BD FACSVerse 3L8C cytometer (San Jose, CA, USA). Data were processed with FlowJo software V10 (BD Biosciences, San Jose, CA, USA). PE-anti-human-CD11b-antibody and FITC-anti-human-CD15 were obtained from Biolegend.

### In vitro neutrophil extracellular traps formation

Primary mouse neutrophils and neutrophil-like differentiated HL-60 cells were seeded onto coverslips pretreated with 100 µg/mL poly-L-lysine at 4 °C overnight in 24-well plates (1 × 10^6^ cells/well) and cultured in serum-free RPMI 1640 medium. Neutrophil extracellular traps were induced with PMA (100 nM, 4 h) in the presence or absence of HVO-rat serum (tenfold dilution with culture medium) or SIV (20 µM), and a negative control was set up.

### Immunofluorescence staining

The culture supernatants were removed, and the cells were washed twice with phosphate-buffered saline. The cells were fixed with 4% paraformaldehyde for 10 min, permeabilized with 0.5% Triton X-100 (Sigma Aldrich) for 15 min, and blocked with 5% BSA in PBS for 1 h at room temperature. Cells were incubated with anti-MPO (1:100; Beyotime) antibody, anti-histone H3 (citrulline R2 + R8 + R17) antibody (1:200; Abcam) and anti-Neutrophil Elastase antibody (1:200; Abcam) at 4 °C overnight, washed with PBS, and incubated with FITC -conjugated goat anti-rabbit IgG (1:200; Abbkine) for 1 h. The washed coverslips were stained with 4′,6-diamidino-2-phenylindole for 4 min and mounted onto slides using mounting media. Fluorescence images were captured using a confocal microscope (Nikon, NIS-Elements 5.4) at 400 × magnification. The integrated densities per image were quantified using the ImageJ software to determine the proportion of NETs formation induced by the neutrophils.

### Statistical analysis

GraphPad Prism version 8.0 was used for depictive statistical analysis. Data were presented as mean ± SEM and analyzed by one-way analysis of variance, followed by Bonferroni post-test to evaluate the diversity between two groups. *P* < 0.05 were considered significance.

## Results

### HVO attenuated AHR, neutrophil-dominated airway inflammation and remodeling, and mucus overproduction in OVA/LPS-induced SRA mice

**Fig. 1:**
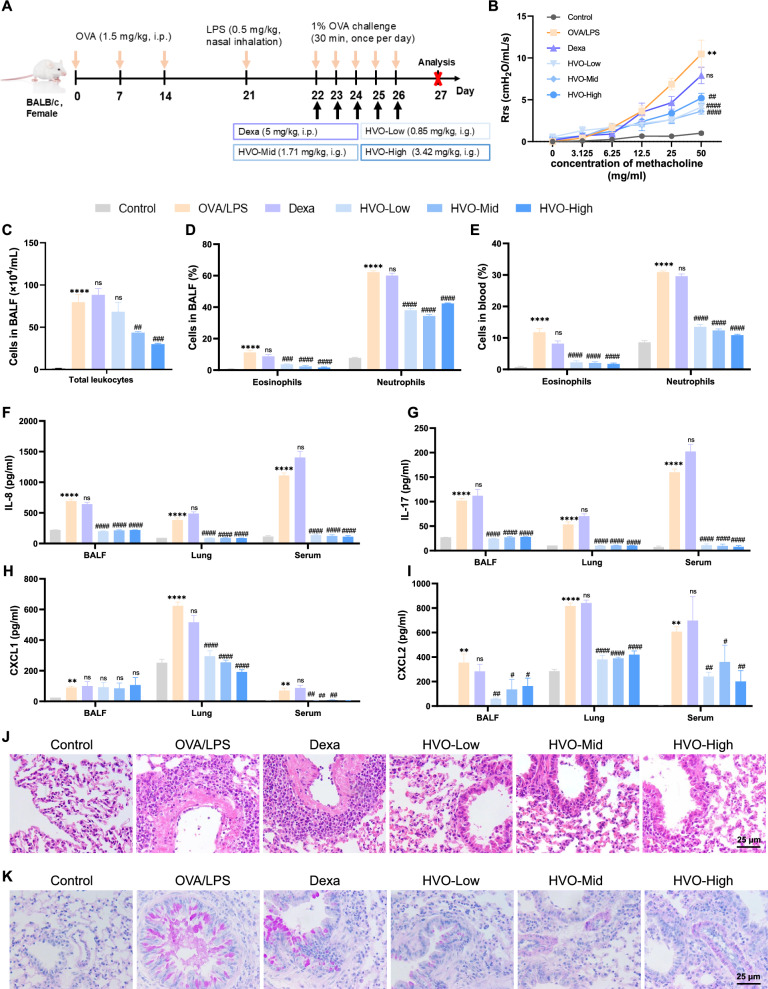
HVO attenuated airway hyperresponsiveness (AHR), airway inflammation and airway remodeling in OVA/LPS-induced SRA mice. **A** Schematic diagram of the SRA mice models induced by OVA/LPS and treated with Dexa or HVO. **B** Evaluation of AHR in response to increasing doses of methacholine. **C** Total leukocytes count in BALF. **D**–**E** Differential count of eosinophils and neutrophils in BALF (**D**) and blood (**E**). **F**–**I** IL-8 (**F**), IL-17 (**G**), CXCL1 (**H**) and CXCL2 (**I**) level in BALF, lungs and serum, measured using ELISA. **J**–**K** Representative images of HE staining (**J**) and PAS staining (**K**) in the lung tissues. Sizes 400 × : Scale bar = 25 µm. Data was presented as mean ± SEM. ****P < 0.0001, **P < 0.01 versus Control group. ####*P* < 0.0001, ###*P* < 0.001, ##*P* < 0.01, #*P* < 0.05, ns *P* > 0.05 versus OVA/LPS group

As shown in Fig. 1B, compared with the control group, the OVA/LPS group showed a marked increase in resistive resistance. Similarly, the number of total leukocytes in BALF (Fig. 1C), neutrophil and eosinophil in both the BALF (Fig. 1D) and blood samples (Fig. 1E) were also increased remarkedly in OVA/LPS group. Furthermore, neutrophils were the main inflammatory cells that induced airway inflammation (an 8.0-fold increase in BALF and a 3.6-fold increase in blood samples). Compared with those in the control group, the OVA/LPS group had a significant increase in the levels of IL-8, IL-17, CXCL1, and CXCL2 in the BALF, lung, and serum samples (Fig. 1F–I). Notable peribranchial inflammatory infiltration, bronchial epithelial mucosal damage, airway goblet cell hyperplasia, mucus hypersecretion, and airway stenosis were also observed in the OVA/LPS group (Fig. 1J–K). However, the effect of dexamethasone was completely abrogated, as indicated by the almost nonexistent suppression of AHR (Fig. 1B), inflammatory cell infiltration (Fig. 1C–J), inflammatory cytokine levels (Fig. 1F–I), and airway remodeling (Fig. 1K). These findings supported our hypothesis that neutrophil-induced airway inflammation in OVA/LPS-induced SRA mice was associated with steroid resistance. Notably, after treatment with HVO, the levels of AHR, inflammatory cell infiltration, and inflammatory cytokine were reduced, and airway remodeling was improved compared with the OVA/LPS group (Fig. 1B–J). Collectively, these results indicated that HVO was able to ameliorate AHR, neutrophil-dominated airway inflammation, and airway remodeling on SRA mice model. Impressively, all dosages of HVO significantly reduced AHR and BALF inflammatory cell infiltration compared with the OVA/LPS group, not in a dose-dependent manner.

### Characterization and identification of compounds in the serum of HVO-treated rats

**Fig. 2:**
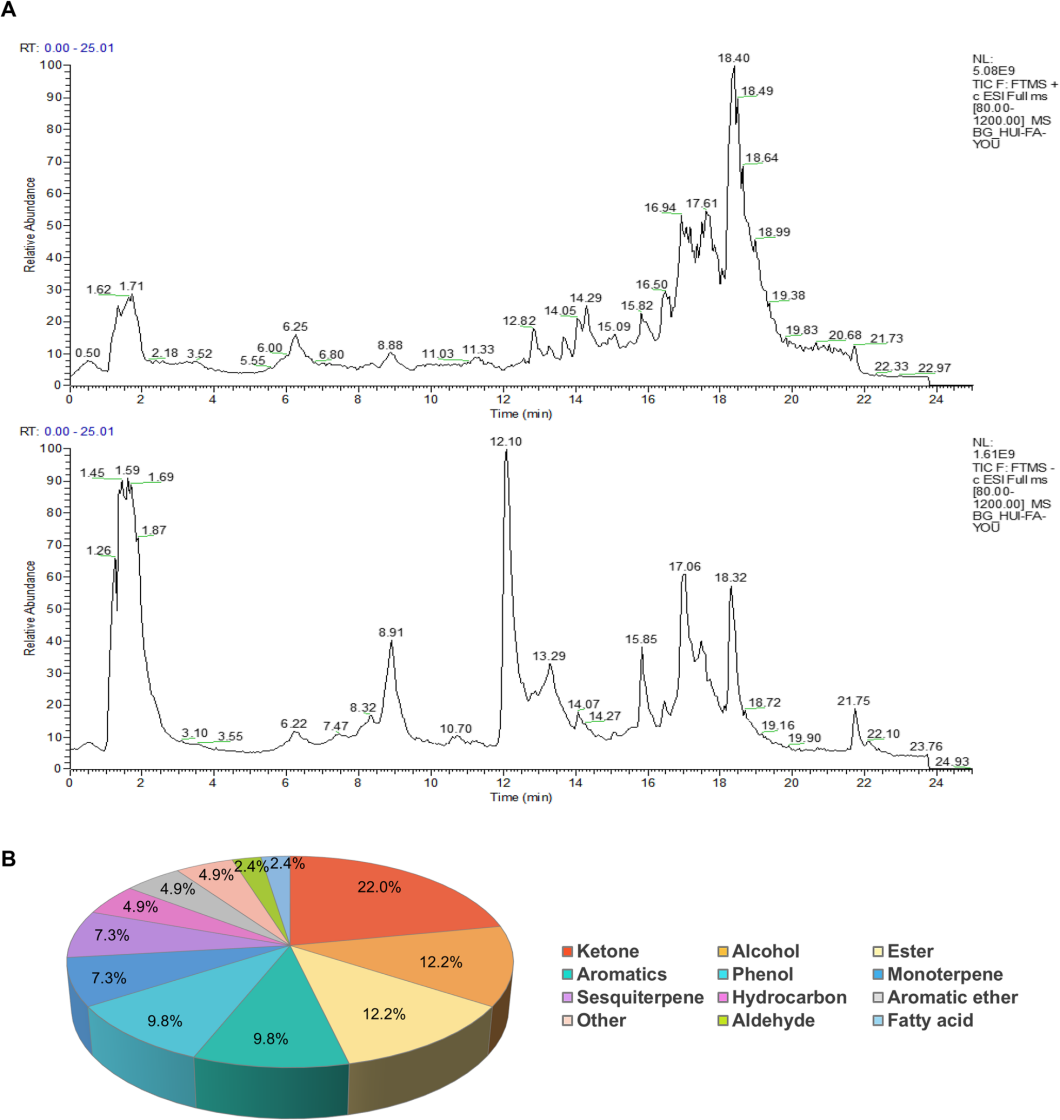
Characterization and identification of in vivo compounds in HVO by UPLC-QE-Orbitrap-MS. **A** Total ion chromatogram (TIC) of rat serum in both positive and negative modes. **B** Structural classification of prototype compounds in serum containing-HVO

Based on the pharmacological effects of HVO on SRA, further studies were conducted to analyze the ingredients in the serum of HVO-treated rats. The ingredients of in vivo protypes, Phase I metabolites, and Phase II metabolites were identified using UPLC-QE-Orbitrap-MS. The total ion chromatogram is shown in Fig. 2A, and the extraction ion chromatograms of characteristic compounds are provided in **Supplementary** Fig. S1. As detailed in **Supplementary** Tables S1–3, the serum contained 41 prototype compounds, 65 Phase I and 50 Phase II metabolites of HVO. The structural types of these 41 prototype compounds are shown in Fig. 2B, with ketone compounds playing a dominant role.

### Network pharmacology analysis of HVO against SRA and prediction of pharmacologically active compounds via molecular docking

**Fig. 3:**
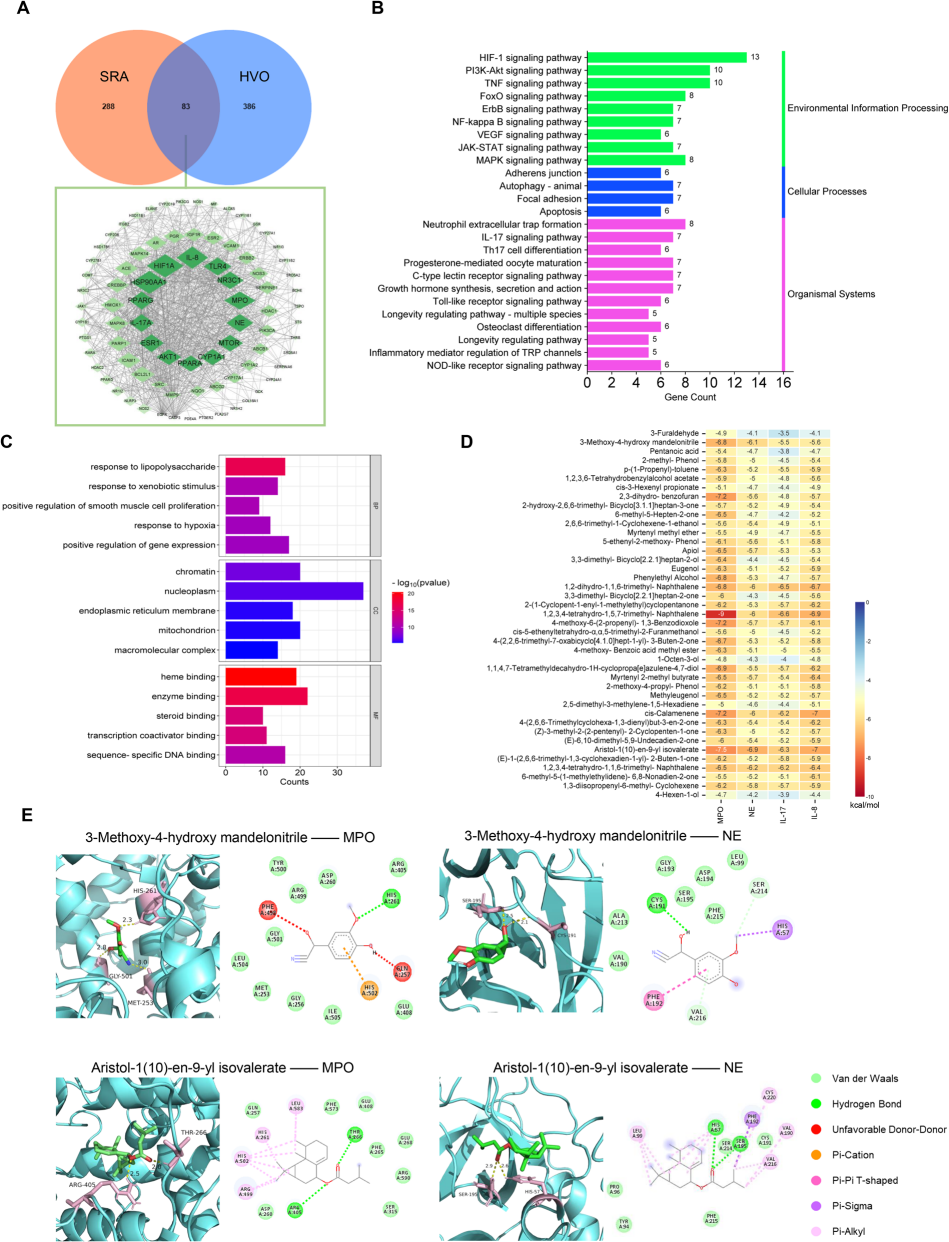
Network topology analysis of HVO against SRA and molecular docking of identified prototype compounds with key targets. **A** Venn Diagram oftargets of prototype compounds in HVO-containing serum and SRA, and PPI core network of the common targets between them. **B** Biological process analysis of overlapping targets was classified by GO annotation. **C** Analysis of KEGG pathway enrichment. **D** The heatmap of docking scores of key targets combining to 41prototype compounds in HVO-containing serum. **E** The representative docking complex of key targets and compounds

Based on the identification of 41 prototype compounds, we used network pharmacology methods to predict the mechanism of HVO against SRA. A total of 471 ingredient-related targets and 371 SRA-associated targets were obtained. In addition, 83 common targets were considered potential targets for HVO and protein-protein interaction network of the potential targets indicated SP90AA, HIF1A, IL-8, TLR4, NR3C1, MPO, NE, MTOR, CYP1A1, PPARA, AKT1, ESR1, IL-17A, and PPARG were key targets with high degree values (Fig. 3A). The results of Kyoto Encyclopedia of Genes and Genomes pathway secondary classification revealed that neutrophil extracellular traps formation may be an important mechanism underlying the effects of HVO on SRA. Additionally, the IL-17 signaling pathway and Th17 cell differentiation were associated with neutrophil-induced immune responses and NETs formation (Fig. 3B). Gene Ontology annotation was conducted, and the results are shown in Fig. 3C. To further explore the active compounds in HVO, molecular docking of 41 prototype compounds was performed with the key targets associated with neutrophil extracellular traps formation and the IL-17 signal pathway, i.e. IL-8, IL-17, MPO, and NE. As shown in Fig. 3D, 3-methoxy-4-hydroxy mandelonitrile, 1,2,3,4-tetrahydro-1,5,7-trimethyl- Naphthalene, cis-calamenene and aristol-1(10)-en-9-yl isovalerate had strong binding affinity with the targets MPO and NE. Figure 3E displays the representative molecular docking results of the most stable pair of each key target and the corresponding component.

### HVO inhibited neutrophil extracellular traps (NETs) formation and IL-17-induced neutrophilic recruitment in OVA/LPS-induced SRA mice

**Fig. 4:**
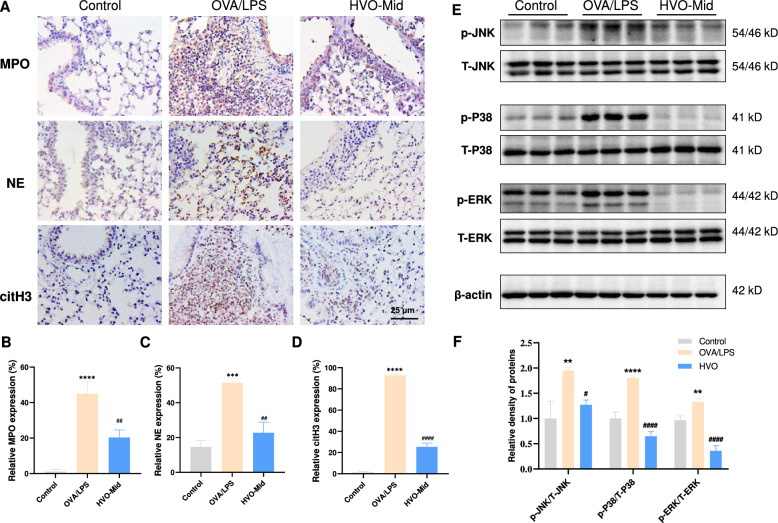
HVO attenuated OVA/LPS-induced neutrophil-dominated airway inflammation by inhibiting NETs formation and IL-17 signaling pathway. **A**–**D** Immunohistochemical representative images **a** in the lung tissues and quantification analysis of the expressions of MPO **B**, NE **C** and citH3 **D** in **A**. Sizes 400 × : Scale bar = 25 µm. **E**–**F** Representative western blot bands **E** of p-JNK, T-JNK, p-P38, T-P38, p-ERK, T-ERK, and β-actin in OVA/LPS-induced mouse lung tissues, and relative phosphorylated protein levels **F** of JNK, P38, and ERK in E. Protein expressions of p-PI3K, p-Akt, p-JNK, and p-P38 in each group. Data was presented as mean ± SEM. ****P < 0.0001, ***P < 0.001, **P < 0.01 versus Control group. ####*P* < 0.0001, ###*P* < 0.001, ##*P* < 0.01, ns *P* > 0.05 versus OVA/LPS group

To confirm the network topology, we performed assays on related proteins implicated in the process of NETs formation. Since NETs are complexes composed of DNA webs and globular proteins including MPO, NE, and citH3 (Fig. S3), [7], the relative levels of MPO, NE, and citH3 in lung tissue were used to evaluate the inhibitory effect of HVO on NETs formation. NETs were found to be upregulated in the OVA/LPS group, evidenced by higher expression of MPO, NE, and citH3 than that in the control group. However, after treatment with HVO, the levels of NETs were reduced (Fig. 4A–D). Concurrently, the IL-17 signaling pathway was enriched according to KEGG analyses (Fig. 3B). The mitogen-activated protein kinases (MAPKs) is recognized to be involved in IL-17 signaling pathway as well as IL-17-induced release of neutrophil-mobilizing cytokines (Fig. S3) [16, 17], proteins associated with the activation of MAPKs (p-ERK, p-JNK and p-P38) were examined in mouse lung tissues. The result suggested that the phosphorylation levels of ERK, JNK and P38 were down-regulated by HVO treatment, indicating HVO could inhibit IL-17-induced neutrophil recruitment by inhibiting MAPKs activation (Fig. 4E-F). Thus, we speculated that HVO, especially at the middle dose, could resolve OVA/LPS-induced SRA by inhibiting NETs release and IL-17 signaling pathway. However, the exact mechanism underlying the inhibition requires further investigation.

### HVO inhibits NETs formation through the suppression of neutrophil elastase in OVA/LPS-induced SRA mice

Neutrophil elastase (NE), which is pivotal in NETs formation, is a proteolytic enzyme stored in the azurophilic granules of neutrophils that processes histones, leading to the release of NETs into the extracellular space [18]. Inhibition of NE activity may provide a viable approach for preventing NETs formation [19]. SIV, a second-generation NE inhibitor [20], was used to ascertain the role of NETs formation in a neutrophilic SRA mice model. We also wondered whether the concurrent treatment of HVO and SIV could enhance the therapeutic potential of HVO.

**Fig. 5:**
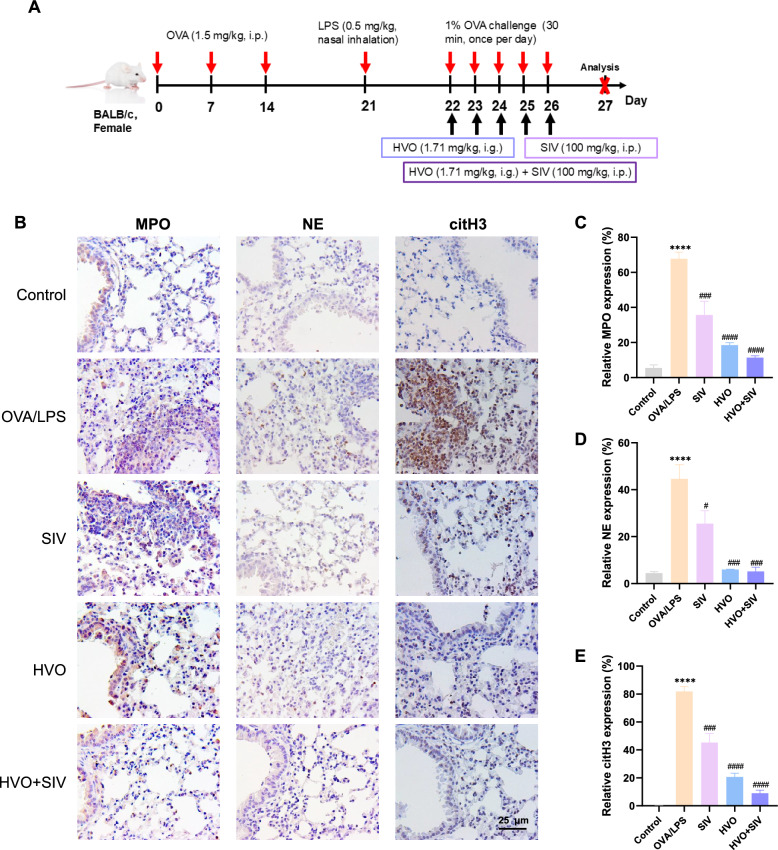
HVO inhibits NETs formation through the suppression of NE in OVA/LPS-induced SRA mice. **A** Schematic diagram of the SRA mice models induced by OVA/LPS and treated with SIV and HVO-Mid (the clinical dosage: 1.71 mg/kg). **B**–**E** Immunohistochemical representative images **B** in the lung tissues and quantification analysis of the expressions of MPO **C**, NE **D** and citH3 **E** in B. Sizes 400 × : Scale bar = 25 µm. Data was presented as mean ± SEM. ****P < 0.0001, **P < 0.01 versus Control group. ####*P* < 0.0001, ###*P* < 0.001, ##*P* < 0.01, ns *P* > 0.05 versus OVA/LPS group

**Fig. 6:**
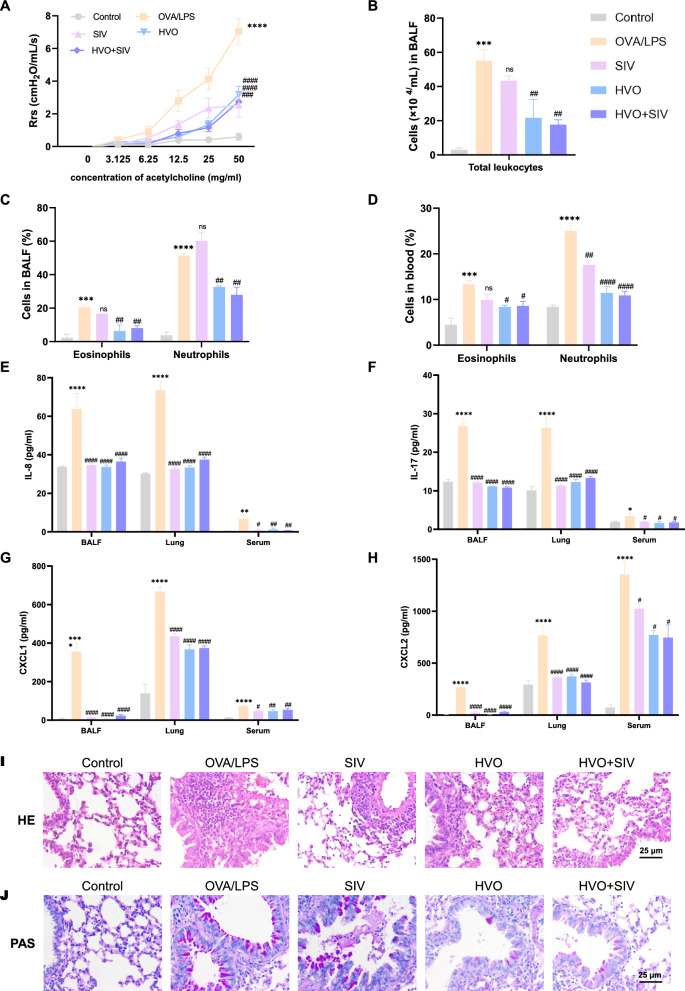
HVO and SIV significantly attenuated airway hyperresponsiveness (AHR), airway inflammation and remodeling in OVA/LPS-induced SRA mice. **A** Evaluation of AHR in response to increasing doses of methacholine. **B** Total leukocytes count in BALF. **C**–**D** Differential count of eosinophils and neutrophils in BALF **C** and blood **D**. **E**–**H** IL-8 **E**, IL-17 **F**, CXCL1 **G** and CXCL2 **H** level in BALF, lungs and serum, measured using ELISA. **I–J** Representative images of HE **I** staining, PAS staining **J** in the lung tissues. Sizes 400 × , Scale bar = 25 µm. Data was presented as mean ± SEM. ****P < 0.0001, ***P < 0.001 versus Control group. ####*P* < 0.0001, ###*P* < 0.001, ##*P* < 0.01, ns *P* > 0.05 versus OVA/LPS group

Using an animal model as shown in Fig. 5A, decreased levels of NE, MPO, and citH3 were observed in the SIV, HVO, and HVO + SIV groups (Fig. 5B–E), indicating that HVO suppressed NETs formation by impeding the release of NE. Simultaneously, SIV or HVO alone attenuated AHR (Fig. 6A), decreased the number of inflammatory cells in the BALF (Fig. 6B, C) and blood (Fig. 6D), reduced the levels of IL-8, IL-17, CXCL1, and CXCL2 (Fig. 6E–H), and alleviated airway inflammation infiltration and airway remodeling (Fig. 6I, J). However, combined treatment with HVO and SIV (HVO + SIV) did not enhance the already suppressed AHR, airway inflammation, and remodeling, and there was no difference compared with HVO or SIV alone (Fig. 6A–J).

Consequently, we believe that HVO inhibits NETs formation by suppressing NE to relieve the symptoms in OVA/LPS-induced SRA mice.

### HVO inhibits phorbol-12-myristate-13-acetate (PMA)-induced NETs formation in vitro

**Fig. 7:**
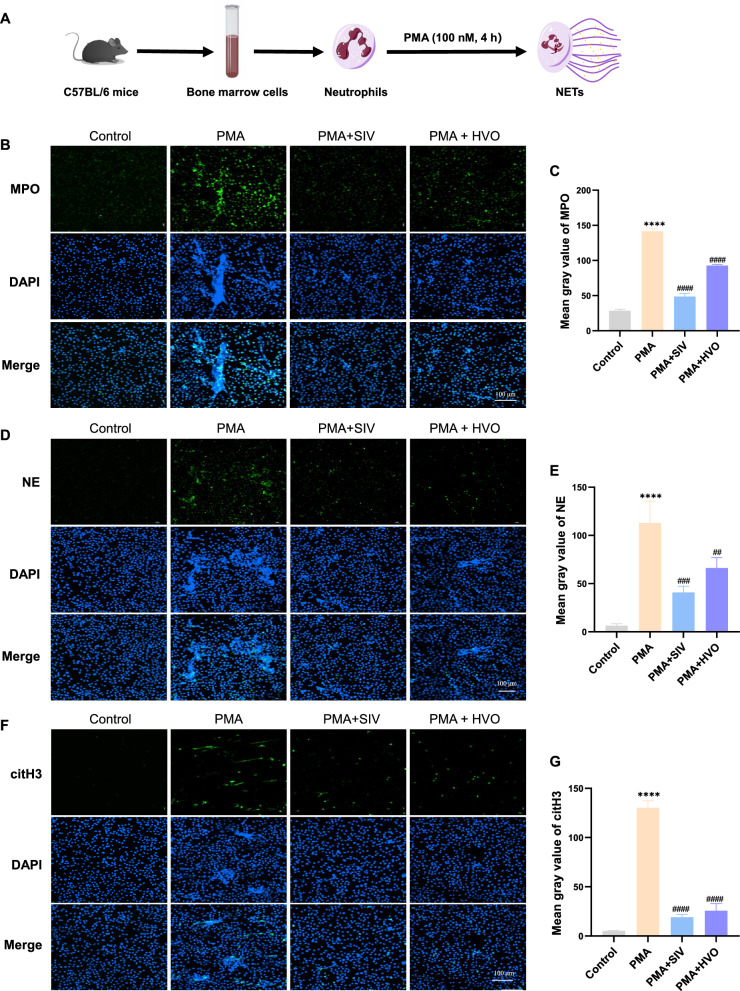
HVO and SIV significantly inhibit PMA-induced NETs formation in mice neutrophils. **A** Schematic diagram showing the isolation of neutrophils from bone marrow cells of C57BL/6 mice in vitro, and their treatment with PMA (100 nM, 4 h) to induce NETs. **B**, **D** and **F** Representative immunofluorescence images of MPO **B**, NE **D** and citH3 **F** in neutrophils isolated from mice bone marrows to visualize NETs formation. Scale bar = 100 µm. **C**, **E** and **G** Quantification of MPO **C**, NE **E** and citH3 **G** and in neutrophils by mean fluorescence intensities. Data was presented as mean ± SEM. ****P < 0.0001, **P < 0.01 versus Control group. ####*P* < 0.0001, ###*P* < 0.001, ##*P* < 0.01 versus PMA group

Next, we evaluated the effect of HVO on NETs formation in vitro. PMA is a classic factor that induces NETs. Bone marrow neutrophils [21] of C57BL/6 mice were isolated (Fig. 7A) and stimulated with PMA to induce NETs formation in the presence or absence of serum containing-HVO or SIV, and then NE, MPO, and citH3 were measured using immunofluorescence staining. As depicted in Fig. 7B–G, PMA-induced NETs formation was significantly decreased by HVO or SIV treatment.

**Fig. 8:**
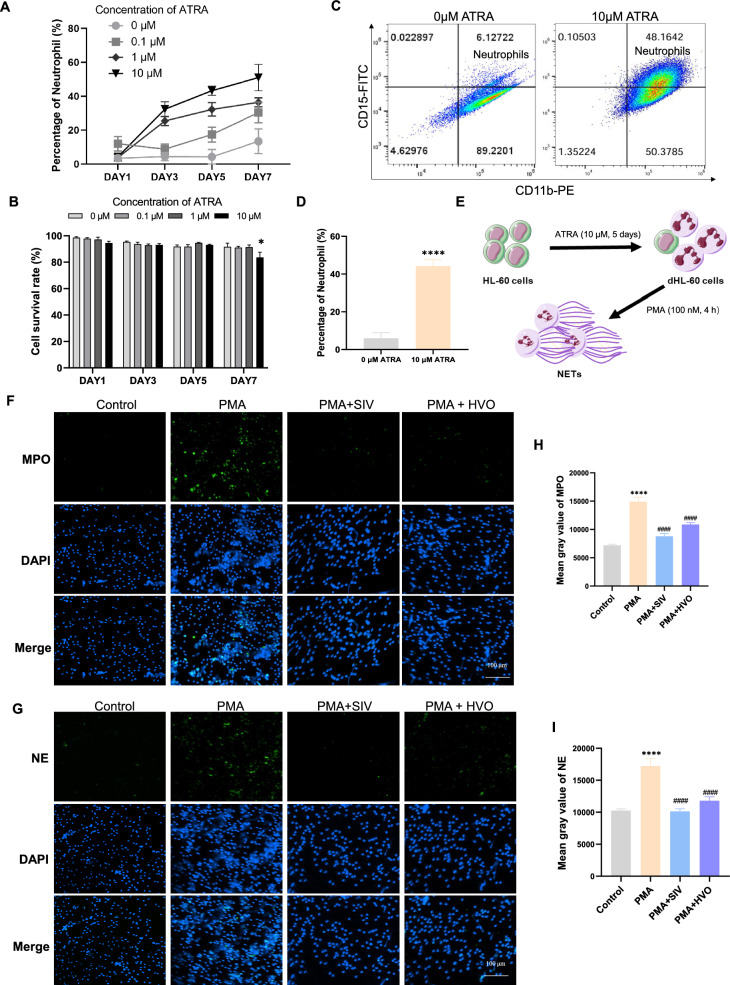
HVO and SIV significantly inhibit PMA-induced NETs formation in human neutrophils. **A** Differential level of HL-60 cells into neutrophil-like dHL-60 cells induced by different concentration of ATRA and different induction time, measured by Giemsa staining. **B** Cell survival rate of dHL-60 cells at different concentration of ATRA and different induction time by trypan blue (0.08%) staining. **C**, **D** Representative flow cytometry plots and quantification of neutrophils (CD11b + CD15 +) differentiated from HL-60 cells with ATRA for 5 days. **e** Schematic diagram showing the differentiation of HL-60 cells to neutrophil-like dHL-60 cells, and their treatment with PMA (100 nM, 4 h) to induce NETs. **F**–**I** Representative immunofluorescence images and quantification of mean fluorescence intensities of MPO **F**, **H** and NE **G**, **I** in neutrophils differentiated from HL-60 cells to visualize NETs formation. Scale bar = 100 µm. Data was presented as mean ± SEM. *P < 0.05 versus 0 μM DAY1. ****P < 0.0001 versus Control group. ####*P* < 0.0001, ###*P* < 0.001, ##*P* < 0.01, ns *P* > 0.05 versus PMA group

To investigate whether human neutrophils could recapitulate these results, HL-60 cells were used [22]. Treatment with 10 µM ATRA for 5 days (more neutrophils and cell survival rate) was the optimal condition for inducing HL-60 cells into neutrophil-like dHL-60 cells (Fig. 8A–D). Subsequently, the effects of HVO and SIV on NETs formation were examined using IF staining. Compared with that in the PMA group, NETs formation was significantly decreased by either HVO or SIV treatment in human neutrophils (Fig. 8F–I).

Collectively, these in vitro experiments confirmed that HVO inhibited PMA-induced NETs formation by suppressing NE.

## Discussion

HVO, an active fraction of the Uygur herb *H. cuspidatus*, has excellent bioactivity and is effective in treating common allergic asthma [23]. The present studies address the question whether HVO have the same therapeutic effects in SRA. As shown in the in vivo experiments, HVO effectively alleviated AHR and inhibited the aggregation of neutrophils around the bronchi, as well as the inflammatory response and airway mucus overproduction caused by neutrophils. This finding suggests that HVO has a therapeutic effect against SRA.

HVO, as a complex mixture, the elucidation of the pharmacologically active constituents is important for its development as a potential treatment for SRA [24]. In this study, we precisely identified 41 prototypes, 65 phase I metabolites and 50 phase II metabolites in HVO-containing serum (**Supplementary** Tables S1–3). Moreover, network pharmacology analysis revealed that the key signaling pathways are intimately linked to the formation of NETs. Particularly, MPO and NE, as the key targets in HVO’s treatment of SRA, play an essential role in the formation of NETs. During the formation of NETs, MPO and NE migrate to the nucleus from azurosome granules, cleave histones, leading to histones citrullination and chromatin decondensation, and subsequently, the plasma membrane ruptures, releasing the web-like NETs [25]. Therefore, we conducted molecular docking experiments of compounds in serum-containing HVO and key targets, by which we discovered that 3-methoxy-4-hydroxy mandelonitrile, 1,2,3,4-tetrahydro-1,5,7-trimethyl-naphthalene, *cis*-calamenene and aristol-1(10)-en-9-yl isovalerate showed excellent affinity for MPO and NE. These compounds above, which can target the key proteins MPO and NE involved in the formation of NETs, may be the therapeutic compounds of HVO in the treatment of SRA, and hold value for further research.

**Fig. 9:**
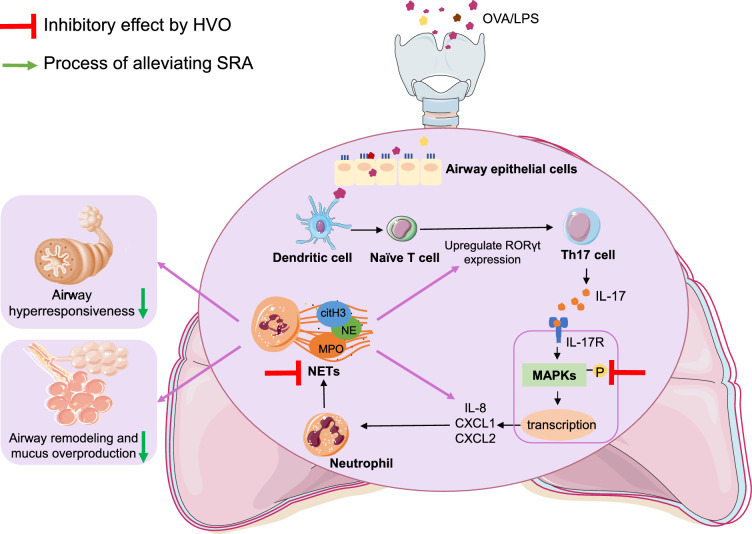
Possible mechanism of the pulmonary protective effects of HVO on SRA

Mechanistic research helps understand how drugs intervene the pathological processes, which is important for the development of new drugs. In this study, the network pharmacology and in vivo*/*in vitro experiments were explored to offer a mechanisms exploration of HVO associated with SRA. NETs, DNA-based webs coated with citH3, and antimicrobial proteins such as NE and MPO, play crucial roles in the innate immune system by trapping and neutralizing pathogens, and several studies have demonstrated their roles in airway inflammation and subsequent airway epithelial cell damage in SRA [4]. Upon exposure to various triggering factors such as allergens, virus and bacterial components, smoking and inflammatory signals, which stimulate differentiation of TH17 cells from naïve T cell, the IL-17 produced from Th17 cells binds to IL-17R and causes activation of three MAPKs cascades. Once activated by the upstream regulators, the MAPKs translocate to the cell nucleus where they modulate the activity of nuclear transcription factors and kinases, which in turn cause the production of neutrophil-mobilizing cytokines such as IL-8, CXCL1 and CXCL2 [7, 8, 17]. Neutrophils recruited to the airway and lung tissues achieve their host defense role by phagocytosing pathogens, secreting their granules full of cytotoxic enzymes, and expelling NETs [26]. During the processes of NETs formation, mediators released from neutrophils such as NE, MPO, and citH3 promote bronchoconstriction, increased mucus production, cause collateral damage to the lungs, and strengthen the development of neutrophil recruitment and steroid resistance [27]. Our findings of elevated expression of MPO, NE and citH3 around the airways of mice with SRA demonstrate that NETs positively correlates with SRA, consistent with the clinical findings of elevated NETs in the sputum of patients [4]. Moreover, HVO could suppress NETs release, together with the activation of p-ERK, p-JNK, p-p38 in IL-17 signaling pathways and reduced production of IL-8, IL-17, CXCL1 and CXCL2, leading to improvement of AHR, mucus production and airway remodeling (Fig. 9).

Several studies have shown that inhibiting NETs formation with NE inhibitors, can reduce lung pathologies and improve pulmonary function [28]. SIV, a specific inhibitor of NE, has demonstrated efficacy in the treatment of respiratory diseases, including acute lung injury or acute respiratory distress syndrome with systemic inflammatory response syndrome [20]. Likewise, our results suggested that both SIV and HVO effectively inhibit NETs formation and prevent the development of SRA in the OVA/LPS-induced mice model as well as in vitro.

Extensive evidence corroborates the association between NETs and a spectrum of pulmonary disorders. For example, NETs had been found impair obstructive lung function in mice and patients with cystic fibrosis [29]. It was reported that NETs formation was increased in the sputum of patients with chronic obstructive pulmonary disease and was associated with disease severity and airway neutrophil function [30]. NETs and NETs-associated dsDNA have also been found to contribute to the pathogenesis of rhinovirus-induced allergic asthma exacerbations and type 2 immune responses in a mouse model [31]. In the present study, our findings add to this body of evidence by showing an association between NETs and SRA, reinforcing the idea that NETs are central to pulmonary disease, and suggest that inhibiting NETs might be a promising approach for treating neutrophilic lung diseases.

To conclude, HVO attenuated AHR, airway inflammation, mucus overproduction and airway remodeling in OVA/LPS-induced SRA mice by suppressing neutrophil filtration and NETs formation. Additionally, the main effective compounds, such as 3-methoxy-4-hydroxy mandelonitrile, 1,2,3,4-tetrahydro-1,5,7-trimethyl-naphthalene, *cis*-calamenene and aristol-1(10)-en-9-yl isovalerate, play vital role in HVO for the treatment of SRA. This finding implies that HVO may be a novel agent for the development of anti-SRA drugs.

## Supplementary Information

Additional file 1

## Data Availability

The datasets supporting the conclusions of this article are included within the article and other related information are available from the corresponding author on reasonable request.

## Abbreviations

SRA: Steroid-resistant asthma
ICS: Inhaled corticosteroids
NETs: Neutrophil extracellular traps
HVO: *Hyssopus cuspidatus* Volatile oil
OVA: Ovalbumin
LPS: Lipopolysaccharide
Dexa: Dexamethasone
SIV: Sivelestat
PMA: Phorbol-12-myristate-13-acetate
AHR: Airway hyperresponsiveness
KEGG: Kyoto encyclopedia of genes and genomes
MPO: Myeloperoxidase
citH3: Citrullinated histone H3
NE: Neutrophil elastase
MAPKs: Mitogen-activated protein kinases
ROS: Reactive oxygen species
DNase: Deoxyribonuclease
MUC5AC: Mucin 5AC
BALF: Bronchoalveolar lavage fluid
IL-8: Interleukin-8
IL-17: Interleukin-17
TNF-α: Tumor necrosis factor-α
IFN-γ: Interferon-γ
CXCL1: Chemokine (C-X-C motif) ligand 1
CXCL2: Chemokine (C-X-C motif) ligand 2
CXCR2: Chemokine (C-X-C motif) receptor
TIC: Total ion chromatogram
EIC: Extraction ion chromatogram
IVC: Individual ventilated cages
SPF: Specific pathogen-free
ESI: Electrospray ionization
ATRA: All-trans-retinoic acid

## References

[1] Holgate ST, Wenzel S, Postma DS, Weiss ST, Renz H, Sly PD. Asthma. Nature Rev Dis Prim. 2015;1(1):15025. 27189668 10.1038/nrdp.2015.25 PMC7096989

[2] Wenzel SE. Asthma phenotypes: the evolution from clinical to molecular approaches. Nat Med. 2012;18(5):716–25. 22561835 10.1038/nm.2678

[3] Alam R, Good J, Rollins D, Verma M, Chu H, Pham T-H, Martin RJ. Airway and serum biochemical correlates of refractory neutrophilic asthma. J Allergy Clin Immunol. 2017;140(4):1004-1014.e1013. 28163052 10.1016/j.jaci.2016.12.963 PMC5540819

[4] Lachowicz-Scroggins ME, Dunican EM, Charbit AR, Raymond W, Looney MR, Peters MC, Gordon ED, Woodruff PG, Lefrançais E, Phillips BR, et al. Extracellular DNA, Neutrophil Extracellular Traps, and Inflammasome Activation in Severe Asthma. Am J Respir Crit Care Med. 2019;199(9):1076–85. 30888839 10.1164/rccm.201810-1869OC PMC6515873

[5] Marshall CL, Hasani K, Mookherjee N. Immunobiology of steroid-unresponsive severe asthma. Front Allergy. 2021;2: 718267. 35387021 10.3389/falgy.2021.718267 PMC8974815

[6] Thiam HR, Wong SL, Wagner DD, Waterman CM. Cellular Mechanisms of NETosis. Annu Rev Cell Dev Biol. 2020;36(1):191–218. 32663035 10.1146/annurev-cellbio-020520-111016 PMC8499668

[7] Papayannopoulos V. Neutrophil extracellular traps in immunity and disease. Nat Rev Immunol. 2017;18(2):134–47. 28990587 10.1038/nri.2017.105

[8] Newcomb DC, Peebles RS. Th17-mediated inflammation in asthma. Curr Opin Immunol. 2013;25(6):755–60. 24035139 10.1016/j.coi.2013.08.002 PMC3855890

[9] Nunes C, Pereira AM, Morais-Almeida M. Asthma costs and social impact. Asthma Res Pract. 2017;3(1):1–11. 28078100 10.1186/s40733-016-0029-3 PMC5219738

[10] Chung KF, Dixey P, Abubakar-Waziri H, Bhavsar P, Patel PH, Guo S, Ji Y. Characteristics, phenotypes, mechanisms and management of severe asthma. Chin Med J. 2022;135(10):1141–55. 35633594 10.1097/CM9.0000000000001990 PMC9337252

[11] Sun X, Hou T, Cheung E. Iu TN-T, Tam VW-H, Chu IM-T, Tsang MS-M, Chan PK-S, Lam CW-K, Wong C-K: anti-inflammatory mechanisms of the novel cytokine interleukin-38 in allergic asthma. Cell Mol Immunol. 2019;17(6):631–46. 31645649 10.1038/s41423-019-0300-7 PMC7264207

[12] Li P, Wang J, Wang C, Cheng L, Ma Q, Li Y, An Y, Dai H, Duan Y, Wang T, et al. Therapeutic effects and mechanisms study of Hanchuan Zupa Granule in a Guinea pig model of cough variant asthma. J Ethnopharmacol. 2021;269: 113719. 33358856 10.1016/j.jep.2020.113719

[13] Sharifi-Rad J, Quispe C, Kumar M, Akram M, Amin M, Iqbal M, Koirala N, Sytar O, Kregiel D, Nicola S, et al. Hyssopus essential oil: an update of its Phytochemistry, Biological Activities, and Safety Profile. Oxid Med Cell Longev. 2022;2022:1–10. 10.1155/2022/8442734 PMC8776447 35069979

[14] Zhang Y-L, Peng H-M, Li J-J, Chen J, Zhang M-R, Wang X, Wang S-Y, Zhu S-Y, Lu J-K, Fang J-B. The volatile oil of Hyssopus cuspidatus Boriss. (HVO) ameliorates OVA-induced allergic asthma via inhibiting PI3K/Akt/JNK/P38 signaling pathway and maintaining airway barrier integrity. J Ethnopharmacol. 2024;334: 118568. 38996949 10.1016/j.jep.2024.118568

[15] Wang J, Wei L, Gao G, Zhu J, Su X, Sun L. Comprehensive investigation of pharmacodyamic material basis of Wikstroemia indica (L.) C. A. Mey by serum pharmacochemistry and bivariate correlation analysis. J Chromatogr B. 2021;1179: 122770. 10.1016/j.jchromb.2021.122770 34325311

[16] Mills KHG. IL-17 and IL-17-producing cells in protection versus pathology. Nat Rev Immunol. 2022;23(1):38–54. 35790881 10.1038/s41577-022-00746-9 PMC9255545

[17] Laan M, Lötvall J, Chung KF, Lindén A. IL-17-induced cytokine release in human bronchial epithelial cells in vitro: role of mitogen-activated protein (MAP) kinases. Br J Pharmacol. 2009;133(1):200–6. 10.1038/sj.bjp.0704063 PMC1572774 11325811

[18] Korkmaz B, Moreau T, Gauthier F. Neutrophil elastase, proteinase 3 and cathepsin G: physicochemical properties, activity and physiopathological functions. Biochimie. 2008;90(2):227–42. 18021746 10.1016/j.biochi.2007.10.009

[19] Metzler Kathleen D, Goosmann C, Lubojemska A, Zychlinsky A, Papayannopoulos V. A myeloperoxidase-containing complex regulates neutrophil elastase release and actin dynamics during NETosis. Cell Rep. 2014;8(3):883–96. 25066128 10.1016/j.celrep.2014.06.044 PMC4471680

[20] Zeiher BG, Artigas A, Vincent J-L, Dmitrienko A, Jackson K, Thompson BT, Bernard G. Neutrophil elastase inhibition in acute lung injury: results of the STRIVE study. Crit Care Med. 2004;32(8):1695–702. 15286546 10.1097/01.ccm.0000133332.48386.85

[21] Zhan X, Wu R, Kong XH, You Y, He K, Sun XY, Huang Y, Chen WX, Duan L. Elevated neutrophil extracellular traps by HBV-mediated S100A9-TLR4/RAGE-ROS cascade facilitate the growth and metastasis of hepatocellular carcinoma. Cancer Commun. 2022;43(2):225–45. 10.1002/cac2.12388 PMC9926958 36346061

[22] Rada B. Neutrophil Extracellular Traps. NADPH Oxid Methods Protoc. 2019;2019:517–28. 10.1007/978-1-4939-9424-3_31 PMC6874304 31172493

[23] Ma X, Ma X, Ma Z, Sun Z, Yu W, Wang J, Li F, Ding J. The Effects of Uygur HerbHyssopus officinalisL. on the Process of Airway Remodeling in Asthmatic Mice. Evid Based Complement Altern Med. 2014;2014:1–7. 10.1155/2014/710870 PMC4212596 25383084

[24] Gu X, Hao D, Xiao P. Research progress of Chinese herbal medicine compounds and their bioactivities: fruitful 2020. Chin Herbal Med. 2022;14(2):171–86. 10.1016/j.chmed.2022.03.004 PMC9476823 36117669

[25] Keir HR, Chalmers JD. Neutrophil extracellular traps in chronic lung disease: implications for pathogenesis and therapy. Eur Respir Rev. 2022;31(163): 210241. 35197267 10.1183/16000617.0241-2021 PMC9488971

[26] Ray A, Kolls JK. Neutrophilic inflammation in asthma and association with disease severity. Trends Immunol. 2017;38(12):942–54. 28784414 10.1016/j.it.2017.07.003 PMC5711587

[27] Krishnamoorthy N, Douda DN, Brüggemann TR, Ricklefs I, Duvall MG, Abdulnour R-EE, Martinod K, Tavares L, Wang X, Cernadas M et al. Neutrophil cytoplasts induce TH17 differentiation and skew inflammation toward neutrophilia in severe asthma. Sci Immunol 2018;3(26):eaao4747. 10.1126/sciimmunol.aao4747 PMC6320225 30076281

[28] Thomas GM, Carbo C, Curtis BR, Martinod K, Mazo IB, Schatzberg D, Cifuni SM, Fuchs TA, von Andrian UH, Hartwig JH, et al. Extracellular DNA traps are associated with the pathogenesis of TRALI in humans and mice. Blood. 2012;119(26):6335–43. 22596262 10.1182/blood-2012-01-405183 PMC3383196

[29] Marcos V, Zhou Z, Yildirim AÖ, Bohla A, Hector A, Vitkov L, Wiedenbauer E-M, Krautgartner WD, Stoiber W, Belohradsky BH, et al. CXCR2 mediates NADPH oxidase–independent neutrophil extracellular trap formation in cystic fibrosis airway inflammation. Nat Med. 2010;16(9):1018–23. 20818377 10.1038/nm.2209

[30] Dicker AJ, Crichton ML, Pumphrey EG, Cassidy AJ, Suarez-Cuartin G, Sibila O, Furrie E, Fong CJ, Ibrahim W, Brady G, et al. Neutrophil extracellular traps are associated with disease severity and microbiota diversity in patients with chronic obstructive pulmonary disease. J Allergy Clin Immunol. 2018;141(1):117–27. 28506850 10.1016/j.jaci.2017.04.022 PMC5751731

[31] Toussaint M, Jackson DJ, Swieboda D, Guedán A, Tsourouktsoglou T-D, Ching YM, Radermecker C, Makrinioti H, Aniscenko J, Bartlett NW, et al. Host DNA released by NETosis promotes rhinovirus-induced type-2 allergic asthma exacerbation. Nat Med. 2017;23(6):681–91. 28459437 10.1038/nm.4332 PMC5821220

