# Supplementary material for: Hyssopus cuspidatus volatile oil: a potential treatment for steroid-resistant asthma via inhibition of neutrophil extracellular traps
Source: Chin Med. 2025 Feb 3;20:17. doi: 10.1186/s13020-025-01069-2 (PMC11792399; doi:10.1186/s13020-025-01069-2)
Supplement: Supplementary file 1 — Additional file 1 [file 13020_2025_1069_MOESM1_ESM.docx]

Supplementary data for

***Hyssopus cuspidatus* Volatile oil: A potential treatment for steroid-resistant asthma via inhibition of neutrophil extracellular traps**

Xu Wang^1, a^, Hui-Ming Peng^2, a^, Meng-Ru Zhang^1, a^, Jing-Jing Li^3, a^, Chuan-Peng Zhao^1^, Ya-Li Zhang^1^,Si-Yu Wang^1^, Si-Ying Zhu^1^, Jian-Kang Lu^1^, Qiang Yin^4^, and Jin-Bo Fang^1, *^

**Authors**

1: School of Pharmacy, Hubei Key Laboratory of Natural Medicinal Chemistry and Resource Evaluation, Tongji Medical College, Huazhong University of Science and Technology, Wuhan 430030, China

2: Department of Anatomy, Tongji Medical College, Huazhong University of Science and Technology, Wuhan, 430030, China

3: Hubei Shizhen Laboratory, School of Basic Medicine, Hubei University of Chinese Medicine, Wuhan 430065, China

4: Xinjiang Uygur Pharmaceutical Co., Ltd, No. 2, Shenyang Street, Urumqi Economic and Technological Development Zone (Toutunhe District), Xinjiang Uygur Autonomous Region, Urumqi 830026, Xinjiang, China

^a^ Xu Wang, H. Peng, M. Zhang and J. Li contributed equally to this study.

***Corresponding author:**

**Jin-Bo Fang**

Address: School of Pharmacy, Hubei Key Laboratory of Natural Medicinal Chemistry and Resource Evaluation, Tongji Medical College, Huazhong University of Science and Technology (HUST), Wuhan 430030, China

E-mail: fangjb@hust.edu.cn

Tel: 0086-27-83692482

Fax: 0086-27-83692482

**
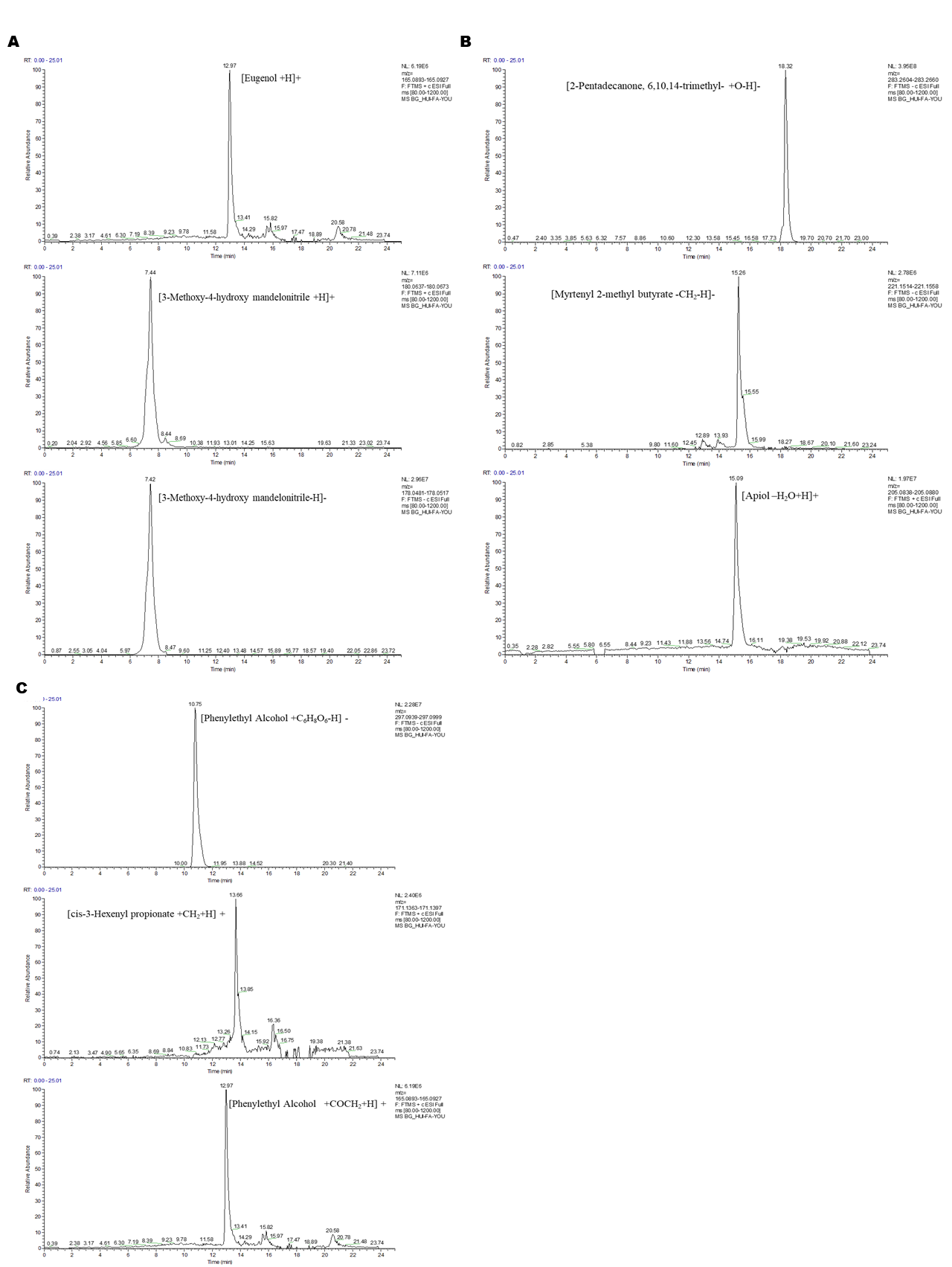
**

**Fig. S1.** Extraction ion chromatogram (EIC) of some characteristic *in vivo* compounds of HVO analyzed by UPLC-QE-Orbitrap-MS.**(A)** EIC of part of prototypes. **(B)** EIC of part of Phase I metabolites. **(C)** EIC of part of Phase II metabolites.

**Table S1**.Characterization of prototypes in rat serum after administration of HVOby UPLC-QE-Orbitrap-MS

| NO. | Identification | Formula | Selected ion | Calculated mass | Measured mass | Error（ppm） | T_R_（min） | MS2 |
| --- | --- | --- | --- | --- | --- | --- | --- | --- |
| 1 | 3-Furaldehyde | C_5_H_4_O_2_ | [M+H^]+^ | 97.0284 | 97.0291 | 6.947 | 1.99 | 97.0289、79.0549、69.0343 |
| 2 | 3-Methoxy-4-hydroxy mandelonitrile | C_9_H_9_NO_3_ | [M-H]^-^  [M+H]^+^ | 178.0499  180.0655 | 178.0504  180.0660 | 2.866  2.445 | 7.42  7.44 | 178.0502、134.0601、121.0284  180.0656、134.9563、105.0340、95.0497 |
| 3 | Pentanoic acid | C_5_H_10_O_2_ | [M+H]^+^ | 103.0754 | 103.0760 | 6.538 | 9.88 |  |
| 4 | 2-methyl- Phenol | C_7_H_8_O | [M-H]^-^ | 107.0491 | 107.0490 | -1.788 | 10.75 | 107.0491、95.8866、63.8480 |
| 5 | p-(1-Propenyl)-toluene | C_10_H_12_ | [M+H]^+^ | 133.1012 | 133.1016 | 3.103 | 10.93 |  |
| 6 | 1,2,3,6-Tetrahydrobenzylalcohol acetate | C_9_H_14_O_2_ | [M-H]^-^ | 153.0910 | 153.0912 | 1.462 | 11.10 | 153.0915、135.0079、109.0283 |
| 7 | cis-3-Hexenyl propionate | C_9_H_16_O_2_ | [M+H]^+^ | 157.1223 | 157.1229 | 3.906 | 12.13 | 157.1221、129.0658、114.0553、97.1017、69.0707 |
| 8 | 2,3-dihydro- benzofuran | C_8_H_8_O | [M-H]^-^ | 119.0491 | 119.0490 | -1.356 | 12.40 | 119.0493、90.0336 |
| 9 | 2-hydroxy-2,6,6-trimethyl- Bicyclo[3.1.1]heptan-3-one | C_10_H_16_O_2_ | [M+H]^+^ | 169.1223 | 169.1228 | 3.097 | 12.42 |  |
| 10 | 6-methyl-5-Hepten-2-one | C_8_H_14_O | [M+H]^+^ | 127.1117 | 127.1123 | 4.393 | 12.47 |  |
| 11 | 2,6,6-trimethyl-1-Cyclohexene-1-ethanol | C_11_H_20_O | [M+H]^+^ | 169.1587 | 169.1591 | 2.354 | 12.57 |  |
| 12 | Myrtenyl methyl ether | C_11_H_18_O | [M+H]^+^ | 167.1430 | 167.1435 | 2.861 | 12.57 | 167.1431、106.9641、79.0549 |
| 13 | 5-ethenyl-2-methoxy- Phenol | C_9_H_10_O_2_ | [M+H]^+^ | 151.0754 | 151.0758 | 2.673 | 12.57 |  |
| 14 | Apiol | C_12_H_14_O_4_ | [M-H]^-^ | 221.0808 | 221.0816 | 3.594 | 12.70 | 221.0820、177.0916、149.0960、134.0361、103.9190、91.9084、71.0489 |
| 15 | 3,3-dimethyl- Bicyclo[2.2.1]heptan-2-ol | C_9_H_16_O | [M+H]^+^ | 141.1274 | 141.1277 | 2.184 | 12.72 |  |
| 16 | Eugenol | C_10_H_12_O_2_ | [M+H]^+^ | 165.0910 | 165.0913 | 2.022 | 12.97 | 165.0911、150.9689、137.0961、122.9743、104.9638 |
| 17 | Phenylethyl Alcohol | C_8_H_10_O | [M-H]^-^ | 121.0648 | 121.0647 | -1.003 | 13.09 | 121.0647、106.0413、94.0284 |
| 18 | 1,2-dihydro-1,1,6-trimethyl- Naphthalene | C_13_H_16_ | [M+H]^+^ | 173.1325 | 173.1330 | 2.847 | 13.26 |  |
| 19 | 3,3-dimethyl- Bicyclo[2.2.1]heptan-2-one | C_9_H_14_O | [M+H]^+^ | 139.1117 | 139.1121 | 2.432 | 13.26 |  |
| 20 | 2-(1-Cyclopent-1-enyl-1-methylethyl)cyclopentanone | C_13_H_20_O | [M+H]^+^ | 193.1587 | 193.1592 | 2.786 | 13.31 | 193.1588、175.1483、147.1169、133.1013、119.0859、109.1016、95.0862 |
| 21 | 1,2,3,4-tetrahydro-1,5,7-trimethyl- Naphthalene | C_13_H_18_ | [M+H]^+^ | 175.1481 | 175.1486 | 2.471 | 13.31 | 175.1482、147.1169、133.1013、105.0705、81.0706 |
| 22 | 4-methoxy-6-(2-propenyl)- 1,3-Benzodioxole | C_11_H_12_O_3_ | [M+H]^+^ | 193.0859 | 193.0864 | 2.689 | 13.56 | 193.1588、175.1482、147.0554、133.1013、109.1016、95.0861 |
| 23 | cis-5-ethenyltetrahydro-α,α,5-trimethyl-2-Furanmethanol | C_10_H_18_O_2_ | [M+H]^+^  [M-H]^-^ | 171.1380  169.1223 | 171.1382  169.1227 | 1.482  2.387 | 13.66  13.83 | 171.1377、139.1118、111.1172、69.0707  169.1226、139.2616、125.0234、113.9250、91.9125 |
| 24 | 4-(2,2,6-trimethyl-7-oxabicyclo[4.1.0]hept-1-yl)- 3-Buten-2-one | C_13_H_20_O_2_  C_13_H_20_O_2_ | [M+H]^+^  [M-H]^-^ | 209.1536  207.1380 | 209.1540  207.1387 | 1.643  3.687 | 13.66  13.68 | 209.1537、152.0706、95.0862  207.1385、165.9991、79.9560 |
| 25 | 4-methoxy- Benzoic acid methyl ester | C_9_H_10_O_3_ | [M+H]^+^ | 167.0703 | 167.0707 | 2.330 | 13.85 |  |
| 26 | 1-Octen-3-ol | C_8_H_16_O | [M+H]^+^ | 129.1274 | 129.1277 | 2.542 | 14.05 |  |
| 27 | 1,1,4,7-Tetramethyldecahydro-1H-cyclopropa[e]azulene-4,7-diol | C_15_H_26_O_2_ | [M-H]^-^ | 237.1849 | 237.1860 | 4.694 | 14.37 | 237.1855、195.1748、168.2530、91.9095 |
| 28 | Myrtenyl 2-methyl butyrate | C_15_H_24_O_2_ | [M+H]^+^ | 237.1849 | 237.1853 | 1.870 | 15.82 | 237.1853、219.1749、201.1640、177.1638、163.1118、149.0962、121.1015、107.0860、95.0861、81.0706 |
| 29 | 2-methoxy-4-propyl- Phenol | C_10_H_14_O_2_ | [M+H]^+^ | 167.1067 | 167.1069 | 1.758 | 15.82 |  |
| 30 | Methyleugenol | C_11_H_14_O_2_ | [M+H]^+^ | 179.1067 | 179.1070 | 1.863 | 15.87 |  |
| 31 | 2,5-dimethyl-3-methylene-1,5-Hexadiene | C_9_H_14_ | [M+H]^+^ | 123.1168 | 123.1172 | 3.435 | 15.87 |  |
| 32 | cis-Calamenene | C_15_H_22_ | [M+H]^+^ | 203.1794 | 203.1799 | 2.277 | 15.97 |  |
| 33 | 4-(2,6,6-Trimethylcyclohexa-1,3-dienyl)but-3-en-2-one | C_13_H_18_O | [M+H]^+^ | 191.1430 | 191.1434 | 1.874 | 16.07 |  |
| 34 | (Z)-3-methyl-2-(2-pentenyl)- 2-Cyclopenten-1-one | C_11_H_16_O | [M+H]^+^ | 165.1274 | 165.1278 | 2.412 | 16.36 |  |
| 35 | (E)-6,10-dimethyl-5,9-Undecadien-2-one | C_13_H_22_O | [M+H]^+^ | 195.1743 | 195.1747 | 2.040 | 16.79 |  |
| 36 | Aristol-1(10)-en-9-yl isovalerate | C_20_H_32_O_2_ | [M-H]^-^ | 303.2319 | 303.2333 | 4.793 | 17.01 | 303.2331、259.2434、205.1957、127.1099、59.0126 |
| 37 | (E)-1-(2,6,6-trimethyl-1,3-cyclohexadien-1-yl)- 2-Buten-1-one | C_13_H_18_O | [M+H]^+^ | 191.1430 | 191.1434 | 2.136 | 17.18 |  |
| 38 | 1,2,3,4-tetrahydro-1,1,6-trimethyl- Naphthalene | C_13_H_18_ | [M+H]^+^ | 175.1481 | 175.1484 | 1.444 | 17.18 | 175.1483、147.1166、133.1014、119.0859 |
| 39 | 6-methyl-5-(1-methylethylidene)- 6,8-Nonadien-2-one | C_13_H_20_O | [M+H]^+^ | 193.1587 | 193.1590 | 1.440 | 17.27 | 193.1590、175.1483、149.1325、133.1013、119.0858、109.1016、95.0862 |
| 40 | 1,3-diisopropenyl-6-methyl- Cyclohexene | C_13_H_20_ | [M+H]^+^ | 177.1638 | 177.1639 | 0.919 | 18.49 |  |
| 41 | 4-Hexen-1-ol | C_6_H_12_O | [M+H]^+^ | 101.0961 | 101.0968 | 7.205 | 21.03 | 101.0967、69.0707、59.0501 |

**Table S2.** Characterization of phase I metabolites in rat serum after administration of HVOby UPLC-QE-Orbitrap-MS.

| NO. | Identification | Formula | Selected ion | Calculated mass | Measured mass | Error（ppm） | T_R_（min） | MS2 |
| --- | --- | --- | --- | --- | --- | --- | --- | --- |
| 1 | 4-Aminophenol | C_6_H_7_NO | [M+H]^+^ | 110.06 | 110.0606 | 4.902 | 1.38 | 110.0607、80.0503 |
| 2 | Hydro 3-Methoxy-4-hydroxy mandelonitrile | C_9_H_11_NO_3_ | [M+H]^+^  [M-H]^-^ | 182.0812  180.0655 | 182.0817  180.066 | 2.912  2.889 | 2.38  2.6 | 182.0815、165.0547、136.0758、123.0443、72.0816  180.0659、163.0393、136.9088、92.9187 |
| 3 | Hydroxyl pentanoic acid | C_5_H_10_O_3_ | [M-H]^-^ | 117.0546 | 117.0546 | 0.08 | 5.82 | 117.0545、71.0489 |
| 4 | Hydro 4-Hydroxybenzoic acid | C_7_H_8_O_3_ | [M+H]^+^ | 141.0546 | 141.0550 | 2.973 | 6.45 | 141.0544、131.9746、113.9641、95.0497 |
| 5 | Hydroxyl 4-methoxy- Benzoic acid methyl ester | C_9_H_10_O_4_ | [M-H]^-^ | 181.0495 | 181.0501 | 3.285 | 6.97 | 181.0499、163.0391、135.0441、119.0491、94.9158、72.9918 |
| 6 | Hydroxyl cis-3-Hexenyl propionate | C_9_H_16_O_3_ | [M+H]^+^ | 173.1172 | 173.1159 | -7.399 | 7.14 |  |
| 7 | Hydroxyl Phenylethyl Alcohol | C_8_H_10_O_2_ | [M+H]^+^ | 139.0754 | 139.0758 | 3.263 | 7.84 |  |
| 8 | Benzoic acid | C_7_H_6_O_2_ | [M-H]^-^ | 121.0284 | 121.0284 | 0.281 | 8.07 | 121.0283、94.0285 |
| 9 | Demethyl 2-methyl- Phenol | C_6_H_6_O | [M-H]^-^ | 93.0335 | 93.0332 | -2.594 | 8.17 | 93.0333、69.2316 |
| 10 | Demethyl and hydro eugenol | C_9_H_12_O_2_ | [M+H]^+^ | 153.0910 | 153.0914 | 2.442 | 9.28 |  |
| 11 | Hydroxyl 5-ethenyl-2-methoxy- Phenol | C_9_H_10_O_3_ | [M-H]^-^ | 165.0546 | 165.055 | 2.359 | 9.45 | 165.0548、147.0442、121.0647、107.0490、72.9917 |
| 12 | Dehydroxyl 3-Methoxy-4-hydroxy mandelonitrile | C_9_H_9_NO_2_ | [M-H]^-^ | 162.055 | 162.0553 | 2.005 | 9.9 | 162.0554、119.0491、73.0282 |
| 13 | 4-nitrophenol | C_6_H_5_NO_3_ | [M-H]^-^ | 138.0186 | 138.0188 | 1.67 | 9.95 | 108.0187、108.0205、93.0333 |
| 14 | Demethyl Phenylethyl Alcohol | C_7_H_8_O | [M-H]^-^ | 107.0491 | 107.049 | -1.788 | 10.75 | 107.0491、95.8866、74.9904、63.8480 |
| 15 | 4-Hydroxybenzoic acid | C_7_H_6_O_3_ | [M-H]^-^ | 137.0233 | 137.0235 | 1.383 | 10.9 | 137.0234、93.0333 |
| 16 | Dehydro cis-3-Hexenyl propionate | C_9_H_14_O_2_ | [M+H]^+^ | 155.1067 | 155.107 | 2.41 | 11.13 |  |
| 17 | Hydroxyl 2-methoxy-4-propyl- Phenol | C_10_H_14_O_3_ | [M+H]^+^ | 183.1016 | 183.1023 | 3.764 | 11.98 | 183.1025、165.0700、137.0962、123.0808、95.0861 |
| 18 | Hydroxyl 6-methyl- 5-Hepten-2-one | C_8_H_14_O_2_ | [M+H]^+^ | 143.1067 | 143.1072 | 3.66 | 12.13 | 143.1068、111.0444、97.1018、73.0656、55.0552 |
| 19 | Hydroxyl 1-Octen-3-ol | C_8_H_16_O_2_ | [M-H]^-^ | 143.1067 | 143.1068 | 1.074 | 12.15 | 143.1068、91.9117 |
| 20 | Hydroxyl styrene | C_8_H_8_O | [M-H]^-^ | 119.0491 | 119.049 | -1.356 | 12.4 | 119.0493、90.0336 |
| 21 | Hydroxyl 4-methoxy-6-(2-propenyl)- 1,3-Benzodioxole | C_11_H_12_O_4_ | [M+H]^+^ | 209.0808 | 209.0815 | 3.179 | 12.52 |  |
| 22 | Hydroxyl (Z)-3-methyl-2-(2-pentenyl)- 2-Cyclopenten-1-one | C_11_H_16_O_2_ | [M+H]^+^ | 181.1223 | 181.1228 | 2.726 | 12.52 |  |
| 23 | Hydroeugenol | C_10_H_14_O_2_ | [M-H]^-^ | 165.091 | 165.0914 | 2.204 | 12.55 | 165.0912、136.9310、121.0282、79.9562 |
| 24 | Dehydro 2,6,6-trimethyl-1-Cyclohexene-1-ethanol | C_11_H_18_O | [M+H]^+^ | 167.143 | 167.1435 | 2.861 | 12.57 | 167.1431、106.9641、79.0549 |
| 25 | Demethyl 6-methyl-5-(1-methylethylidene)- 6,8-Nonadien-2-one | C_12_H_18_O | [M+H]^+^ | 179.143 | 179.1435 | 2.614 | 12.57 | 179.1432、161.1326、150.0268、133.1013、95.0861 |
| 26 | Demethyleugenol | C_9_H_10_O_2_ | [M+H]^+^ | 151.0754 | 151.0758 | 2.673 | 12.57 |  |
| 27 | 4-methoxybenzoic acid | C_8_H_8_O_3_ | [M-H]^-^ | 151.039 | 151.0392 | 1.32 | 12.89 | 151.0392、107.0491、66.2647 |
| 28 | Dehydro 2-methoxy-4-propyl- Phenol | C_10_H_12_O_2_ | [M+H]^+^ | 165.091 | 165.0913 | 2.022 | 12.97 | 165.0911、137.0961、122.9743、104.9638 |
| 29 | Hydro 2,3-dihydro- Benzofuran | C_8_H_10_O | [M-H]^-^ | 121.0648 | 121.0647 | -1.003 | 13.09 | 121.0647、106.0413、94.0284 |
| 30 | Hydro 4-methoxy-6-(2-propenyl)- 1,3-Benzodioxole | C_11_H_14_O_3_ | [M-H]^-^ | 193.0859 | 193.0866 | 3.414 | 13.19 |  |
| 31 | Hydroxyl 2,5-dimethyl-3-methylene-1,5-Hexadiene | C_9_H_14_O | [M+H]^+^ | 139.1117 | 139.1121 | 2.432 | 13.26 |  |
| 32 | Hydroxyl (E)-6,10-dimethyl-5,9-Undecadien-2-one | C_13_H_22_O_2_ | [M+H]^+^ | 211.1693 | 211.1697 | 2.29 | 13.31 | 211.1690、193.1587、175.1484、133.1015、109.1019、95.0861 |
| 33 | Hydroxyl trimethyl-2-hydroxy-2,6,6- Bicyclo[3.1.1]heptan-3-one | C_10_H_16_O_3_ | [M+H]^+^ | 185.1172 | 185.1177 | 2.642 | 13.31 |  |
| 34 | Hydro 1,2-dihydro-1,1,6-trimethyl- Naphthalene | C_13_H_18_ | [M+H]^+^ | 175.1481 | 175.1486 | 2.471 | 13.31 | 175.1482、133.1013、119.0858、105.0705 |
| 35 | Dehydro (E)-6,10-dimethyl-5,9-Undecadien-2-one | C_13_H_20_O | [M+H]^+^ | 193.1587 | 193.1592 | 2.786 | 13.31 | 193.1588、175.1483、147.1169、133.1013、119.0859、109.1016、95.0862 |
| 36 | Hydro cis-3-Hexenyl propionate | C_9_H_18_O_2_ | [M-H]^-^ | 157.1223 | 157.1226 | 1.615 | 13.34 | 157.1225、89.0229 |
| 37 | Hydro 1,2,3,6-Tetrahydrobenzylalcohol acetate | C_9_H_16_O_2_ | [M+H]^+^ | 157.1223 | 157.1227 | 2.697 | 13.36 |  |
| 38 | Hydroxyl 1,2,3,6-Tetrahydrobenzylalcohol acetate | C_9_H_14_O_3_ | [M-H]^-^ | 169.0859 | 169.0862 | 1.651 | 13.38 | 169.0862、125.0960、91.9080 |
| 39 | Hydroxyl linalool | C_10_H_18_O_2_ | [M+H]^+^  [M-H]^-^ | 171.138  169.1223 | 171.1382  169.1227 | 1.482  2.387 | 13.66  13.83 | 171.1377、139.1118、111.1172、69.0707  169.1226、125.0234、113.9250 |
| 40 | Hydroxyl 6-methyl-5-(1-methylethylidene)- 6,8-Nonadien-2-one | C_13_H_20_O_2_ | [M+H]^+^  [M-H]^-^ | 209.1536  207.139 | 209.1539  207.1387 | 1.643  3.687 | 13.66  13.68 | 209.1537、152.0706、95.0862  207.1385、165.9991、79.9560 |
| 41 | Hydro cis- 5-ethenyltetrahydro-α,α,5-trimethyl-2-Furanmethanol | C_10_H_20_O_2_ | [M-H]^-^ | 171.138 | 171.1384 | 2.651 | 13.68 | 171.1380、127.9561、115.9195、99.9246 |
| 42 | Hydroxyl methyleugenol | C_11_H_14_O_3_ | [M+H]^+^ | 195.1016 | 195.1021 | 2.917 | 13.85 |  |
| 43 | Hydroxyl myrtenyl 2-methyl butyrate | C_15_H_24_O_3_ | [M+H]^+^ | 253.1798 | 253.1803 | 2.049 | 13.95 |  |
| 44 | Hydroxyl (1S-cis)-1,2,3,4-tetrahydro-1,6-dimethyl-4-(1-methylethyl)- Naphthalene | C_15_H_22_O | [M+H]^+^ | 219.1743 | 219.1748 | 1.953 | 14 |  |
| 45 | Hydro 6-methyl-5-Hepten-2-one | C_8_H_16_O | [M+H]^+^ | 129.1274 | 129.1277 | 2.542 | 14.05 |  |
| 46 | Demethyl and dedihydroxyl eugenol | C9H10 | [M+H]^+^ | 119.0855 | 119.0858 | 2.293 | 14.10 | 119.0857、91.0848 |
| 47 | Hydroxyl p-(1-Propenyl)-toluene | C_10_H_12_O | [M+H]^+^ | 149.0961 | 149.0964 | 1.8 | 14.10 |  |
| 48 | Dehydroxyl 5-ethenyl-2-methoxy- Phenol | C_9_H_10_O | [M+H]^+^ | 135.0804 | 135.0806 | 1.247 | 14.10 | 135.0806、107.0860、95.0497 |
| 49 | Hydroxyl 2,6,6-trimethyl-1-Cyclohexene-1-ethanol | C_11_H_20_O_2_ | [M+H]^+^ | 185.1536 | 185.154 | 2.072 | 14.15 | 185.1538、157.1225、139.1118、111.1173、97.1018、69.0707 |
| 50 | Hydroxyl 2,3-dihydro- Benzofuran | C_8_H_8_O_2_ | [M+H]^+^ | 137.0597 | 137.0601 | 3.02 | 14.74 |  |
| 51 | Dehydromyrtenyl 2-methyl butyrate | C_15_H_22_O_2_ | [M+H]^+^  [M-H]^-^ | 235.1693  233.1536 | 235.1699  233.1547 | 2.779  4.733 | 14.74  14.76 | 235.1692、172.1154、116.0531、89.0425  233.1544、99.9244 |
| 52 | Hydroxyl 3-benzyloxy-2-fluoro-4-methoxy- benzaldehyde | C_15_H_13_FO_4_ | [M+H]^+^ | 277.087 | 277.0866 | 2.596 | 14.84 |  |
| 53 | Hydro methyleugenol | C_11_H_16_O_2_ | [M+H]^+^ | 181.1223 | 181.1221 | -1.36 | 14.94 | 181.1224、163.0390、149.0235 |
| 54 | Dehydrated apiol | C_12_H_12_O_3_ | [M+H]^+^ | 205.0859 | 205.0864 | 2.337 | 15.09 | 167.0339、149.0235、121.0285、57.0708 |
| 55 | Demethylmyrtenyl 2-methyl butyrate | C_14_H_22_O_2_ | [M-H]^-^ | 221.1536 | 221.1546 | 4.312 | 15.26 | 221.1543、177.1275、91.9122、 |
| 56 | Hydroxyl eugenol | C_10_H_12_O_3_ | [M+H]^+^ | 181.0859 | 181.0863 | 2.094 | 15.43 | 149.0235、107.0496、91.8904 |
| 57 | Dehydro 2-methyl- Phenol | C_7_H_6_O | [M+H]^+^ | 107.0491 | 107.0497 | 5.592 | 15.43 | 107.0496、93.0579 |
| 58 | Dehydrophenacylidene diacetate | C_12_H_10_O_5_ | [M+H]^+^ | 235.0601 | 235.0593 | -3.275 | 15.48 | 235.0589、179.1067、57.0708 |
| 59 | Dehydroxylmethyleugenol |  | [M+H]^+^ | 163.1117 | 163.112 | 1.645 | 15.48 | 163.1117、133.0286、105.0340 |
| 60 | Demethyl [1ar-(1aα,4aα,7β,7aβ,7bα)]^-^ decahydro-1,1,7-trimethyl-4-methylene-1H-Cycloprop[e]azulen-7-ol | C_14_H_22_O | [M-H]^-^ | 205.1587 | 205.1595 | 3.939 | 15.6 | 205.1594、165.9993、129.6441、79.9561 |
| 61 | Hydroxyl (1S,3aR,4R,8R,8aS)-1-Isopropyl-3a-methyl-7-methylenedecahydro-4,8-epoxyazulene | C_15_H_24_O_2_ | [M+H]^+^ | 237.1849 | 237.1853 | 1.87 | 15.82 | 237.1853、177.1638、149.0962、121.1015、81.0706 |
| 62 | Dehydro p-(1-Propenyl)-toluene | C_10_H_10_ | [M+H]^+^ | 131.0855 | 131.0859 | 2.541 | 15.82 |  |
| 63 | Dehydro (Z)- 3-methyl-2-(2-pentenyl)- 2-Cyclopenten-1-one | C_11_H_14_O | [M+H]^+^ | 163.1117 | 163.112 | 1.645 | 15.87 | 163.1117、105.0340 |
| 64 | Dehydro 6,10,14-trimethyl-2-Pentadecanone | C_18_H_34_O | [M+H]^+^ | 267.2682 | 267.2685 | 0.927 | 17.61 | 267.2688、171.9105、123.1172、109.1017、95.0861、81.0706、71.0863、57.0708 |
| 65 | Hydroxyl 6,10,14-trimethyl-2-Pentadecanone | C_18_H_36_O_2_ | [M-H]^-^ | 283.2632 | 283.2646 | 5.059 | 18.32 | 283.2644、99.4546、75.8600 |

**Table S3**. Characterization of phase II metabolites in rat serum after administration of HVO.

| NO. | Identification | Formula | Selected ion | Calculated mass | Measured mass | Error（ppm） | T_R_（min） | MS2 |
| --- | --- | --- | --- | --- | --- | --- | --- | --- |
| 1 | Methyl 4-Aminophenol | C_7_H_9_NO | [M+H]^+^ | 124.0757 | 124.0761 | 3.300 | 1.38 | 124.0759、107.0735、94.0656、79.0551 |
| 2 | Acetylated pyrrole | C_6_H_7_NO | [M+H]^+^ | 110.0600 | 110.0606 | 4.902 | 1.38 | 110.0607、80.0503 |
| 3 | Dimethyl 4-Aminophenol | C_8_H_11_NO | [M+H]^+^ | 138.0913 | 138.0916 | 2.096 | 1.43 | 138.0914、120.0811、94.0658、80.9486 |
| 4 | Trimethyl 4-Aminophenol | C_9_H_13_NO | [M+H]^+^ | 152.1070 | 152.1073 | 2.034 | 1.62 | 152.1071、134.0964、121.0889、94.0657、76.0763 |
| 5 | Methyl pyridine | C_6_H_7_N | [M+H]^+^ | 94.0651 | 94.0659 | 7.911 | 3.12 | 94.0657、67.0551、62.9908 |
| 6 | Glucuronide-conjugated 1-Octen-3-ol | C_14_H_24_O_7_ | [M+H]^+^ | 305.1595 | 305.1579 | -5.078 | 7.49 | 305.1570、248.1755、163.1231、58.0660 |
| 7 | Methyl 3-Methoxy-4-hydroxy mandelonitrile | C_10_H_11_NO_3_ | [M-H]^-^  [M+H]^+^ | 192.0655  194.0812 | 192.0662  194.0818 | 3.438  3.247 | 8.12  8.14 | 192.0661、102.9476、74.0234  194.0813、120.0811、91.0548、76.0401 |
| 8 | Glucuronide-conjugated 2-methyl- Phenol | C_13_H_16_O_7_ | [M-H]^-^ | 283.0812 | 283.0826 | 4.984 | 9.25 | 283.0826、265.0701、175.0239、113.0232、85.0282 |
| 9 | Sulfate-conjugated 4-methoxy- Benzoic acid methyl ester | C_9_H_10_O_6_S | [M-H]^-^ | 245.0114 | 245.0126 | 4.877 | 9.70 | 245.0124、203.0821、165.0549、116.0342、74.0234 |
| 10 | Methyl-3-Furaldehyde | C_6_H_6_O_2_ | [M+H]^+^ | 111.0441 | 111.0446 | 5.169 | 10.48 | 111.0446、83.0498、79.0219、55.0552 |
| 11 | Glucuronide-conjugated 3-Benzyloxy-4-methoxybenzaldehyde | C_21_H_22_O_9_ | [M-H]^-^ | 417.1180 | 417.1200 | 4.678 | 10.60 | 417.1197、348.6211、241.0869、175.0240、113.0233、85.0282 |
| 12 | Glucuronide-conjugated 2-hydroxy-2,6,6-trimethyl- Bicyclo[3.1.1]heptan-3-one | C_16_H_24_O_8_ | [M-H]^-^ | 343.1387 | 343.1403 | 4.418 | 10.70 | 343.1403、297.1328、193.0350、163.0605、113.0232、75.0074 |
| 13 | Glucuronide-conjugated Phenylethyl Alcohol | C_14_H_18_O_7_ | [M-H]^-^ | 297.0969 | 297.0984 | 5.186 | 10.75 | 297.0983、279.0873、219.5849、175.0240、113.0232、85.0282 |
| 14 | Sulfate-conjugated 2-methyl- Phenol | C_7_H_8_O_4_S | [M-H]^-^ | 187.0060 | 187.0066 | 3.498 | 10.75 | 187.0065、142.1228、107.0490、79.9561 |
| 15 | Acetylated 3-Furaldehyde | C_7_H_6_O_3_ | [M-H]^-^ | 137.0233 | 137.0235 | 1.383 | 10.90 | 137.0234、93.0333 |
| 16 | Trimethyl and hydro 4-Hydroxybenzoic acid | C_10_H_14_O_3_ | [M+H]^+^ | 183.1016 | 183.1023 | 3.764 | 11.98 | 183.1025、123.0808、95.0861 |
| 17 | Acetylated 4-Hexen-1-ol | C_8_H_14_O_2_ | [M+H]^+^ | 143.1067 | 143.1072 | 3.660 | 12.13 | 143.1068、111.0444、97.1018、73.0656、55.0552 |
| 18 | Acetylated 2-hydroxy-2,6,6-trimethyl- Bicyclo[3.1.1]heptan-3-one | C_12_H_18_O_3_ | [M-H]^-^ | 209.1172 | 209.1180 | 3.869 | 12.15 | 209.1180、168.0154、79.9561、59.0125 |
| 19 | Acetylated (-)-Myrtenol | C_12_H_18_O_2_ | [M+H]^+^ | 195.1380 | 195.1386 | 3.196 | 12.18 | 195.1384、135.1170、107.0860、93.0705 |
| 20 | Sulfate-conjugated 2,3-dihydro- Benzofuran | C_8_H_8_O_4_S | [M-H]^-^ | 199.0060 | 199.0066 | 3.186 | 12.25 | 199.0065、156.1385、119.0491、79.9561 |
| 21 | Sulfate-conjugated 2-methoxy-4-propyl- Phenol | C_10_H_14_O_5_S | [M-H]^-^ | 245.0478 | 245.0489 | 4.241 | 12.35 | 245.0489、187.0959、165.0912、143.1068、79.9561 |
| 22 | Acetylated cis- 5-ethenyltetrahydro-α,α,5-trimethyl-2-Furanmethanol | C_12_H_20_O_3_ | [M-H]^-^ | 211.1329 | 211.1337 | 3.690 | 12.50 | 211.1335、167.0493、132.0444、80.9639 |
| 23 | Methyl (Z)- 3-methyl-2-(2-pentenyl)- 2-Cyclopenten-1-one | C_12_H_18_O | [M+H]^+^ | 179.1430 | 179.1435 | 2.614 | 12.57 | 179.1432、161.1326、150.0268、133.1013、119.0859、95.0861、81.0707 |
| 24 | Acetylated linalool | C_12_H_20_O_2_ | [M+H]^+^ | 197.1536 | 197.1542 | 3.112 | 12.57 | 197.1536、161.1327、119.0858、95.0861 |
| 25 | Methyl 5-ethenyl-2-methoxy- Phenol | C_10_H_12_O_2_ | [M+H]^+^ | 165.0910 | 165.0913 | 2.022 | 12.97 | 165.0911、137.0961、122.9743、104.9638、67.0550 |
| 26 | Methyl 2-methyl- phenol | C_8_H_10_O | [M-H]^-^ | 121.0648 | 121.0647 | -1.003 | 13.09 | 121.0647、106.0413、94.0284 |
| 27 | Sulfate-conjugated phenylethyl alcohol | C_8_H_10_O_4_S | [M-H]^-^ | 201.0216 | 201.0224 | 3.701 | 13.09 | 201.0222、157.1225、121.0647、79.9561 |
| 28 | Sulfate-conjugated 3-Benzyloxy-4-methoxybenzaldehyde | C_15_H_14_O_6_S | [M-H]^-^ | 321.0427 | 321.1442 | 4.438 | 13.14 | 321.0440、305.4681、241.0867、201.0224、135.0442、121.0284 |
| 29 | Acetylated 2,6,6-trimethyl-1-Cyclohexene-1-ethanol | C_13_H_22_O_2_ | [M+H]^+^ | 211.1693 | 211.1697 | 2.290 | 13.31 | 211.1690、193.1587、175.1484、133.1015、109.1016、95.0861 |
| 30 | Acetylated 1,2,3,6-Tetrahydrobenzylalcohol acetate | C_11_H_16_O_3_ | [M-H]^-^ | 195.1016 | 195.1022 | 3.481 | 13.38 | 195.1030、177.0919、151.1120、91.9118、69.0330 |
| 31 | Methyl cis-3-Hexenyl propionate | C_10_H_18_O_2_ | [M+H]^+^  [M-H]^-^ | 171.1380  169.1223 | 171.1382  169.1227 | 1.482  2.387 | 13.66  13.83 | 171.1377、139.1118、111.1172、97.1017、69.0707  169.1226、125.0234、113.9250、91.9125 |
| 32 | Acetylated myrtenyl methyl ether | C_13_H_20_O_2_ | [M+H]^+^  [M-H]^-^ | 209.1536  207.1380 | 209.1539  207.1387 | 1.643  3.687 | 13.66  13.68 | 209.1537、152.0706、95.0862  207.1385、165.9991、109.0280、79.9560 |
| 33 | Methyl 2,3-dihydro- Benzofuran | C_9_H_10_O | [M+H]^+^ | 135.0804 | 135.0806 | 1.247 | 14.10 | 135.0806、107.0860、95.0497 |
| 34 | Methyl cis-5-ethenyltetrahydro-α,α,5-trimethyl-2-Furanmethanol | C_11_H_20_O_2_ | [M-H]^-^ | 185.1536 | 185.1540 | 2.072 | 14.15 | 185.1538、157.1225、139.1118、111.1173、69.0707 |
| 35 | Acetylated methyleugenol | C_13_H_16_O_3_ | [M+H]^+^ | 221.1172 | 221.1177 | 2.076 | 14.69 | 221.1174、179.1068、57.0708 |
| 36 | Benzoic acid methyl ester | C_8_H_8_O_2_ | [M+H]^+^ | 137.0597 | 137.0601 | 3.02 | 14.74 |  |
| 37 | Acetylated myrtenyl 2-methyl butyrate | C_17_H_26_O_3_ | [M+H]^+^  [M-H]^-^ | 279.1955  277.1798 | 279.1962  277.1813 | 2.503  5.227 | 14.74  14.76 | 279.1954、223.0754、195.0806、149.0234  277.1810、233.1902、158.0960、91.9088、59.0125 |
| 38 | Acetylated 2-(1-Cyclopent-1-enyl-1-methylethyl)cyclopentanone | C_15_H_22_O_2_ | [M+H]^+^  [M-H]^-^ | 235.1693  233.1536 | 235.1699  233.1547 | 2.779  4.733 | 14.74  14.76 | 235.1692、172.1154、116.0531、89.0425  233.1544、99.9244 |
| 39 | Acetylated (1S,3aR,4R,8R,8aS)-1-Isopropyl-3a-methyl-7-methylenedecahydro-4,8-epoxyazulene | C_17_H_26_O_2_ | [M+H]^+^  [M-H]^-^ | 263.2006  261.1849 | 263.2012  261.1863 | 2.330  5.488 | 15.09  15.11 | 263.2006、245.1903、193.1224、133.1015  261.1865、217.1596、163.1483、91.9102 |
| 40 | Methyl 4-methoxy- Benzoic acid methyl ester | C_10_H_12_O_3_ | [M+H]^+^ | 181.0859 | 181.0863 | 2.094 | 15.43 | 163.0390、149.0235、107.0496、91.8904 |
| 41 | Glucuronide-conjugated4-methyl-1-(1-methylethyl)- [1S-(1α,4β,5α)]^-^Bicyclo[3.1.0]hexan-3-one | C_16_H_24_O_7_ | [M+H]^+^ | 329.1595 | 329.1602 | 2.250 | 15.58 | 259.1544、217.0343、185.0810、157.0133、129.0184、61.0293 |
| 42 | Methyl 2-(1-Cyclopent-1-enyl-1-methylethyl)cyclopentanone | C_14_H_22_O | [M-H]^-^ | 205.1587 | 205.1595 | 3.939 | 15.60 | 205.1594、165.9993、129.6441、79.9561 |
| 43 | Methyl methyleugenol | C_12_H_16_O_2_ | [M+H]^+^ | 193.1223 | 193.1227 | 1.883 | 15.82 |  |
| 44 | Acetylated (E)- 6,10-dimethyl-5,9-Undecadien-2-one | C_15_H_24_O_2_ | [M+H]^+^ | 237.1849 | 237.1853 | 1.870 | 15.82 | 237.1853、177.1638、149.0962、121.1015、81.0706 |
| 45 | Methyl myrtenyl 2-methyl butyrate | C_16_H_26_O_2_ | [M-H]^-^ | 249.1849 | 249.1861 | 4.830 | 16.34 | 249.1857、205.1963、162.5447、112.9844 |
| 46 | Acetylated 6,10,14-trimethyl-2-Pentadecanone | C_20_H_38_O_2_ | [M-H]^-^ | 309.2788 | 309.2801 | 4.343 | 18.32 | 309.2800、240.0256、172.5297、96.9588 |
| 47 | Sulfate-conjugated p-Cymen-7-ol | C_10_H_14_O_4_S | [M-H]^-^ | 229.0529 | 229.0538 | 3.772 | 18.42 | 229.0536、196.9939、149.0961、116.9278、79.9561 |
| 48 | Sulfate-conjugated carvone | C_10_H_14_O_4_S | [M-H]^-^ | 229.0529 | 229.0539 | 4.165 | 20.70 | 229.0536、196.9933、149.0961、91.9130、79.9561 |
| 49 | Methyl-1-Penten-3-ol | C_6_H_12_O | [M+H]^+^ | 101.0961 | 101.0968 | 7.205 | 21.03 | 101.0967、69.0707、59.0501 |
| 50 | Sulfate-conjugated pinocarvone | C_10_H_14_O_4_S | [M-H]^-^ | 229.0529 | 229.0539 | 4.514 | 21.75 | 229.0539、196.9936、149.0962、79.9561 |


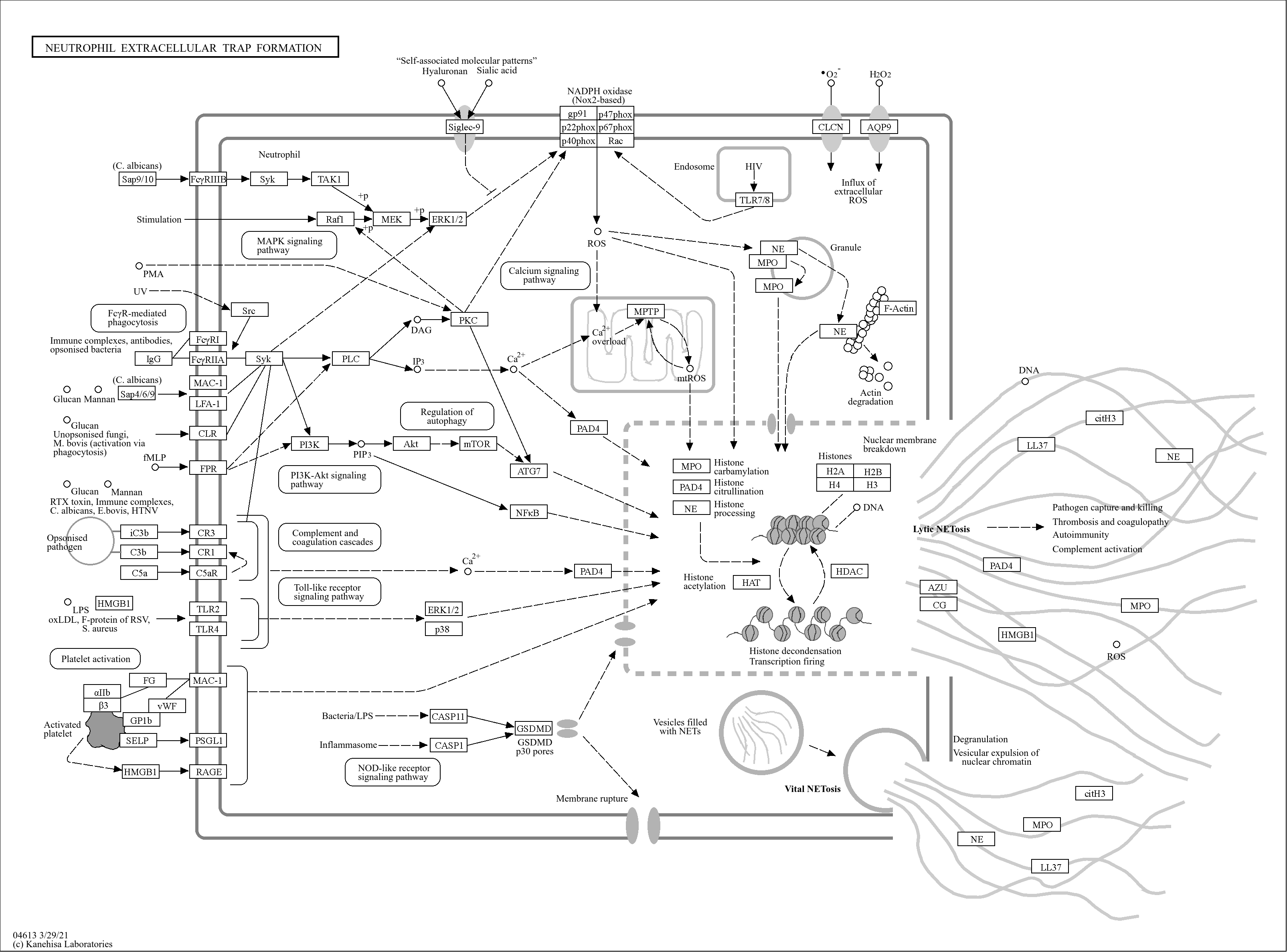


**Fig. S2.**The biological process of Neutrophil Extracellular Traps (NETs) formation by KEGG pathways enrichmentin DAVID database.


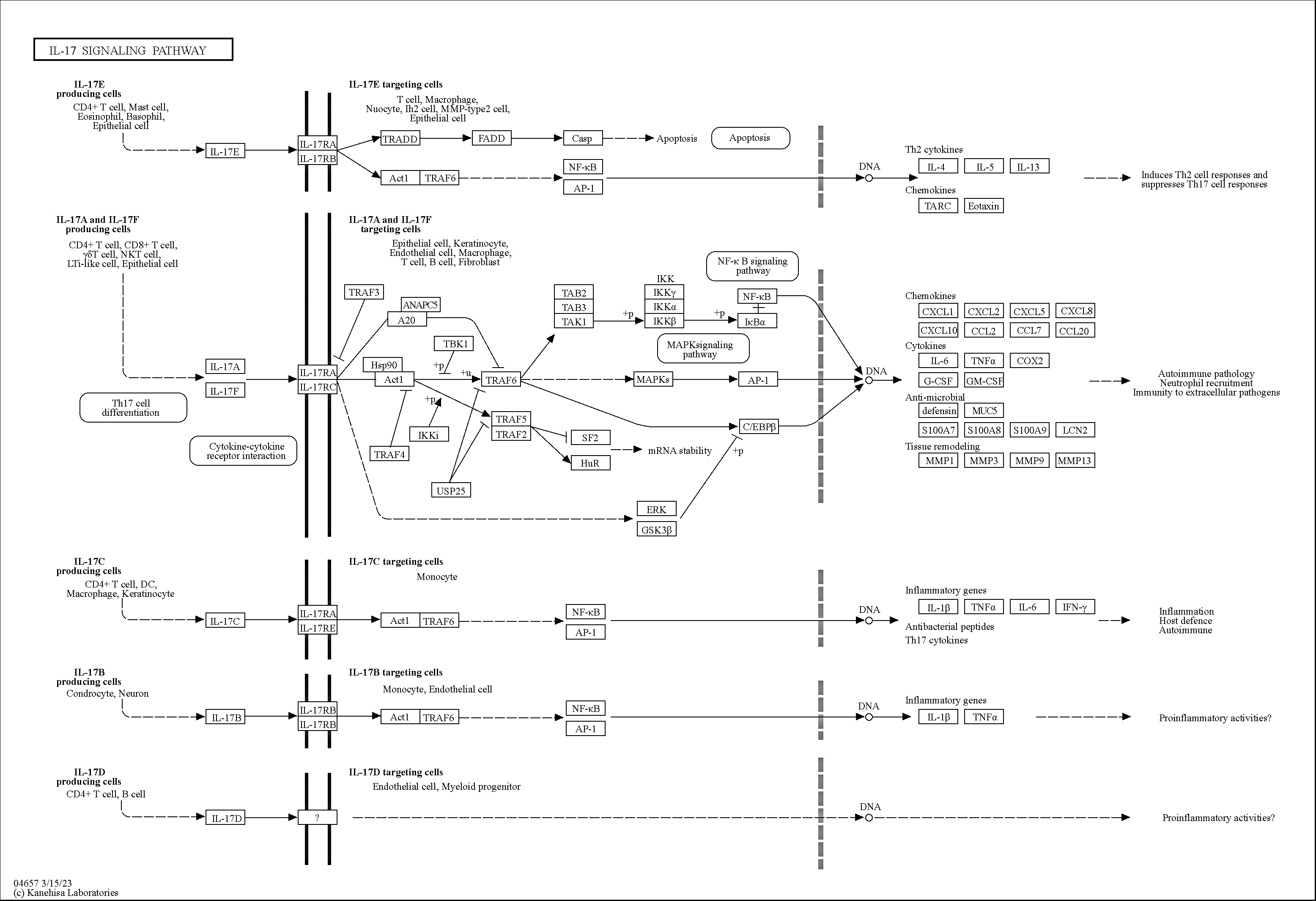


**Fig. S3.**The biological process of IL-17 signaling pathway by KEGG pathways enrichmentin DAVID database. The cytokines and related proteins measured in this studywere marked by red boxes.


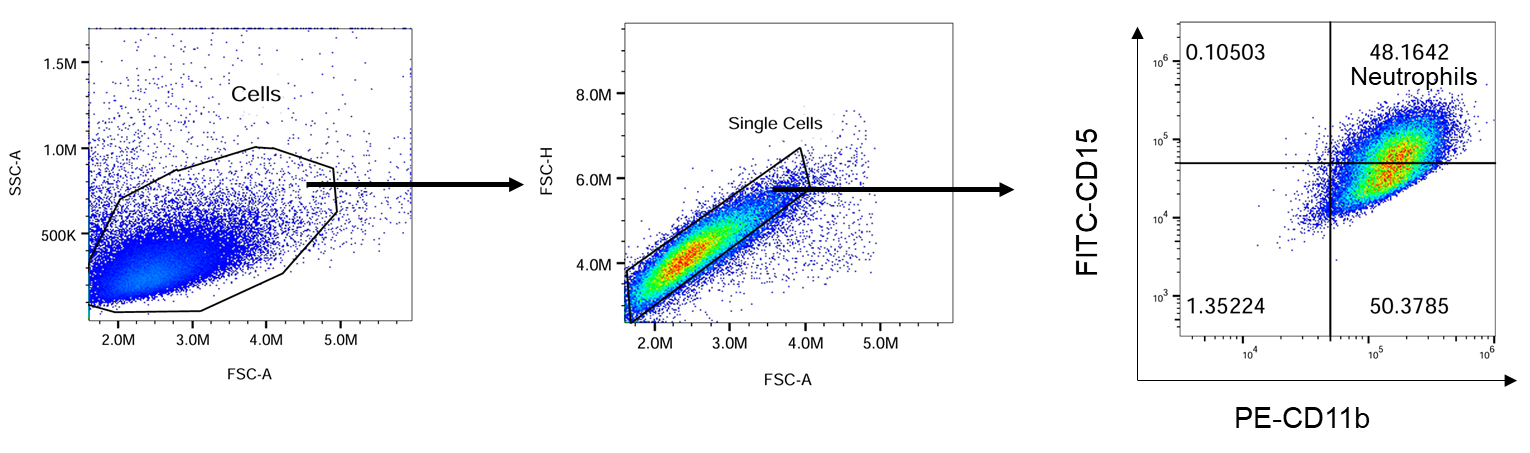


**Fig. S4.** Gating strategy for differentiated HL-60 cells.
